# Vitamin D Signaling through Induction of Paneth Cell Defensins Maintains Gut Microbiota and Improves Metabolic Disorders and Hepatic Steatosis in Animal Models

**DOI:** 10.3389/fphys.2016.00498

**Published:** 2016-11-15

**Authors:** Danmei Su, Yuanyang Nie, Airu Zhu, Zishuo Chen, Pengfei Wu, Li Zhang, Mei Luo, Qun Sun, Linbi Cai, Yuchen Lai, Zhixiong Xiao, Zhongping Duan, Sujun Zheng, Guihui Wu, Richard Hu, Hidekazu Tsukamoto, Aurelia Lugea, Zhenqui Liu, Stephen J. Pandol, Yuan-Ping Han

**Affiliations:** ^1^The Center for Growth, Metabolism and Aging, and the Key Laboratory for Bio-Resource and Eco-Environment of Education of Ministry, College of Life Sciences, Sichuan UniversityChengdu, China; ^2^Chengdu Public Health Clinical CenterChengdu, China; ^3^Beijing YouAn Hospital, Capital Medical UniversityBeijing, China; ^4^Olive View-University of California, Los Angeles Medical CenterLos Angeles, CA, USA; ^5^Department of Pathology, Keck School of Medicine of the University of Southern CaliforniaLos Angeles, CA, USA; ^6^Cedars-Sinai Medical CenterLos Angeles, CA, USA

**Keywords:** vitamin D, metabolic syndrome, non-alcoholic fatty liver disease (NAFLD), non-alcoholic steatohepatitis (NASH), gut microbiota, defensins, helicobacter, *Akkermansia muciniphila*

## Abstract

Metabolic syndrome (MetS), characterized as obesity, insulin resistance, and non-alcoholic fatty liver diseases (NAFLD), is associated with vitamin D insufficiency/deficiency in epidemiological studies, while the underlying mechanism is poorly addressed. On the other hand, disorder of gut microbiota, namely dysbiosis, is known to cause MetS and NAFLD. It is also known that systemic inflammation blocks insulin signaling pathways, leading to insulin resistance and glucose intolerance, which are the driving force for hepatic steatosis. Vitamin D receptor (VDR) is highly expressed in the ileum of the small intestine, which prompted us to test a hypothesis that vitamin D signaling may determine the enterotype of gut microbiota through regulating the intestinal interface. Here, we demonstrate that high-fat-diet feeding (HFD) is necessary but not sufficient, while additional vitamin D deficiency (VDD) as a second hit is needed, to induce robust insulin resistance and fatty liver. Under the two hits (HFD+VDD), the Paneth cell-specific alpha-defensins including α-defensin 5 (DEFA5), MMP7 which activates the pro-defensins, as well as tight junction genes, and MUC2 are all suppressed in the ileum, resulting in mucosal collapse, increased gut permeability, dysbiosis, endotoxemia, systemic inflammation which underlie insulin resistance and hepatic steatosis. Moreover, under the vitamin D deficient high fat feeding (HFD+VDD), *Helicobacter hepaticus*, a known murine hepatic-pathogen, is substantially amplified in the ileum, while *Akkermansia muciniphila*, a beneficial symbiotic, is diminished. Likewise, the VD receptor (VDR) knockout mice exhibit similar phenotypes, showing down regulation of alpha-defensins and MMP7 in the ileum, increased *Helicobacter hepaticus* and suppressed *Akkermansia muciniphila*. Remarkably, oral administration of DEFA5 restored eubiosys, showing suppression of *Helicobacter hepaticus* and increase of *Akkermansia muciniphila* in association with resolving metabolic disorders and fatty liver in the HFD+VDD mice. An *in vitro* analysis showed that DEFA5 peptide could directly suppress *Helicobacter hepaticus*. Thus, the results of this study reveal critical roles of a vitamin D/VDR axis in optimal expression of defensins and tight junction genes in support of intestinal integrity and eubiosis to suppress NAFLD and metabolic disorders.

## Introduction

Metabolic syndrome (MetS) is becoming increasingly prevalent worldwide, and in many countries about 15–20% population bears metabolic disorders (Ford, 2005). Although clinically characterized and diagnosed as different diseases, MetS, non-alcoholic fatty liver disease (NAFLD), and type-II diabetes (T2D) share the common causes and biogenesis events. A “two hit theory” has been proposed to explain the transition from simple steatosis to non-alcoholic steatohepatitis (NASH), characterized by persistent hepatic inflammation, fibrosis and increased risk for cirrhosis or/and liver cancer (Day and James, 1998). On the other hand, insulin resistance (IR) underlies the foundation of systemic metabolic complications, such as hyperglycemia, dyslipidemia, hyperinsulinemia, and diabetes (Shoelson et al., 2006). Evidence exists showing a causal role of chronic low-grade inflammation in development of IR and NASH (Biddinger and Kahn, 2006; Cani et al., 2007). The cellular basis of IR is mediated in part through impairment of insulin signaling pathways, caused by pro-inflammatory cytokines produced in inflamed tissues including visceral fat (Hotamisligil et al., 1993).

Gut microbiota exist in a symbiotic relationship with the host, and have critical roles in development of systemic immunity and protecting the host from colonization of pathogenic microbes (Ley et al., 2005; Brown et al., 2012). Particular enterotypes of gut microbiota are determined or influenced by multiple factors including their maternal source, host genomic polymorphisms, immune status, and dietary composition (Rawls et al., 2006; Nicholson et al., 2012). Unbalanced gut microbiota (dysbiosis), on the other hand, may lead to alteration of immunity and increased risk of diverse diseases, including type-2 diabetes and obesity (Bäckhed et al., 2004; Ley et al., 2005; Larsen et al., 2010). Dysbiosis is often associated with loss of the integrity of intestinal mucosa, which may result in gut impairment and consequent endotoxemia, hepatic bacterial translocation, low-grade systemic inflammation, all of which may drive pathogenesis of insulin resistance, which consequently cause hepatic steatosis and MetS (Cani et al., 2007; Creely et al., 2007; Pendyala et al., 2012).

On the other hand, due to air pollution, insufficient sunlight exposure, and altered dietary composition, vitamin D deficiency (VDD) or insufficiency affects 30–60% of population worldwide, and is increasingly found in association with many diseases including autoimmune diseases, hepatitis, and cancer (Holick et al., 2011). Addition to its classical roles in promoting calcium and phosphorus adsorption, vitamin D in its active format as calcitriol functions like sterol hormones to regulate diverse biological functions from the host immune response to cell differentiation (Adams and Hewison, 2010). Although, high calorie diet including high fat diet (HFD) is thought to be a major cause of IR, NAFLD, and MetS, the epidemiologic evidence also shows an association of VDD in development of MetS (Botella-Carretero et al., 2007; Lu et al., 2009; Barchetta et al., 2011; Bea et al., 2015). In animal experiments, dietary vitamin D deficiency was found to exacerbate Toll-like receptor activation and hepatic inflammation in obese rats (Roth et al., 2012). In a mouse model, we previously demonstrated that vitamin D-deficient-high-fat diet (HFD+VDD) hampers the enterohepatic circulation of bile acids, leading to NASH (Kong et al., 2014). Furthermore, we recently found that long-term dietary vitamin D depletion could generate spontaneous liver fibrosis in a mice model (Zhu et al., 2015). However, the causal role of vitamin D deficiency in the pathogenesis of NAFLD and its underlying mechanism remain largely unknown.

In present study, we investigated the mechanistic roles of vitamin D signaling in maintaining the “gut interface,” namely the interplay between the intestinal epithelium and the adjacent microbiota in the ileum. We found that in presence of sufficient vitamin D supplement (standard AIN93 diet), the mice were tolerant to HFD feeding, showing relatively less insulin resistance and moderate hepatic steatosis. In contrast, feeding the mice with a vitamin D-deficient-high-fat diet (HFD+VDD) led to overt insulin resistance, hepatic steatosis and even signs of NASH. The insulin resistance under the double hits was closely related to a sequential chain of events that start from the VDD-mediated down regulation of ileal Paneth cell specific α-defensins (DEFA), their converting enzyme MMP7, and tight junction genes, to increase of endotoxemia and systemic inflammation, and consequent insulin resistance. Moreover, the HFD-initiated primary dysbiosis was exacerbated by additional VDD, showing overgrowth of the *Helicobacter hepaticus*, a known hepatic pathogen, and suppression of the symbiotic species *Akkermansia muciniphila. In vitro* treatment with human DEFA5 peptide inhibited *H. hepaticus* growth. Oral administration of DEFA5 *in vivo* to the HFD+VDD mice restored ileal eubiosis and intestinal epithelial integrity, and insulin sensitivity. Similar to what we observed in the dietary impact, the genetic ablation of the vitamin D receptor (VDR KO) resulted in reduced DEFA5 and MMP7 expression in the ileum, increased intestinal permeability, ileal dysbiosis, and hepatic steatosis, which collectively demonstrates a critical role for vitamin D signaling in maintaining intestinal integrity, eubiosis, and metabolic homeostasis.

## Materials and methods

### Animals and treatment

All animal experiments in this study were complied with the guidelines from “Guide for the Care and Use of Laboratory Animals” published by the USA National Institutes of Health, and the procedures were approved by The Institutional Animal Care and Use Committee (IACUC) in Sichuan University. Briefly, 4–6 weeks old BALB/c male mice (Beijing HFK Bioscience) were maintained in a controlled environment (12:12 light-dark cycle) with free access to food and water. The mice were fed for 18–20 weeks by four types of diet (*n* = 20 for each condition): (1) control chow with VD_3_ at 1000 IU/kg (standard AIN93 formula), C; (2) vitamin D depleted control chow, VDD; (3) high fat diet (60% calorie from fat) with VD_3_ supplement at 1000 IU/kg, HFD; (4) high fat chow without vitamin D supplement, HFD+VDD. The compositions of the four types of diet and vitamin kit are listed in Tables 1, 2, respectively. Food intake and body weight were recorded weekly. For one experiment, the mice fed with HFD+VDD for 18 weeks were given synthetic human α-defensin-5 (DEFA5, PDF-4415, Peptides International) via oral gavage (10 μg/dose in 0.1 ml saline) for four doses equally spaced over 25 days, or saline as a vehicle control. As another control, a synthetic mutant DEFA5, by which the conserved cysteines and lysines were replaced by alanine and serine, respectively, was also administered in an equal amount via gavage. During the treatment the mice were maintained on HFD+VDD diet; and in additional 10 days the mice were euthanized for measurements. To determine the responsiveness of ileum to vitamin D challenge, the male BALB/c mice fed under vitamin D depletion for 1 month were treated with Rocaltrol (Roche, 5 ng/g body weight) through intraperitoneal injection; and the mice were sacrificed at 0, 6, 12, and 24 h, respectively. VDR knockout mice (B6.129S4-Vdrtm1Mbd/J, the Jackson Laboratory) were fed with a high phosphorus high calcium diet (2% calcium, 1.25% phosphorus) since post-weaning. The VDR^−/−^ (VDR KO) mice and the littermates at the age of 5 month old were sacrificed for measurement. As a standard procedure, all the mice were fasted for 12 h before sacrifice. Under anesthesia, blood was collected and centrifuged to obtain plasma, then stored at −20°C for further measurement. The liver, pancreas, adipose, ileum at the distal region, and ileal lumen microbe contents were harvested and immersed in liquid nitrogen and stored at −80°C for further analysis.

**Table 1:** **Composition of four types of diet used in the study**.

**Ingredient**	**Control (g/kg)**	**VDD (g/kg)**	**HFD (g/kg)**	**HFD+VDD (g/kg)**
Amino acids	195.6	195.6	238.8	238.8
Cornstarch	397.5	397.5	0	0
Dextrinized cornstarch	132	132	179.7	179.7
Sucrose	100	100	92.6	92.6
Fiber	50	50	66.3	66.3
Soybean oil (no additives)	70	70	33.1	33.1
Lard	0	0	319.7	319.7
Mineral mix	35	35	46.4	46.4
Vitamin mix	10 (VD_3_ 1000 IU/kg)	10 (−VD_3_)	10 (VD_3_ 1000 IU/kg)	10 (−VD_3_)
NaHCO_3_	7.4	7.4	9.8	9.8
Choline bitartrate (41.1% choline)	2.5	2.5	3.3	3.3
Tert-butylhydroquinon	0.1	0.1	0.1	0.1
Total	1000	1000	1000	1000

**Table 2:** **Composition of vitamin kit**.

**Vitamin**	**mg or U/kg diet**
Nicotinic acid, mg	30
Pantothenate, mg	15
Pyridoxine, mg	6
Thiamin, mg	5
Riboflavin, mg	6
Folic acid, mg	2
Vitamin K, mg	750
D-Biotin, mg	200
Vitamin B-12, mg	25
Vitamin A, IU	4000
Vitamin D_3_, IU	1000
Vitamin E, IU	75

### Glucose tolerance test (GTTs), insulin tolerance test (ITTs), and HOMA-IR analysis

Mice were fasted for 6 or 4 h for GTTs or ITTs, respectively. Glucose (1 g/kg body weight, 20% glucose solution) or insulin (1 U/kg body weight) was injected intraperitoneally. Blood glucose was tested by a glucose meter (Accu-Chek Active, Roche) with 5 μL blood was collected from the tip of the tail vein. Plasma insulin concentration was measured by ELISA kit (DRE30417, RB China). HOMA-IR = (glucose conc. × insulin con.)/22.5.

### Plasma biochemistry

The plasma LPS concentration was determined by Limulus Amebocyte Extract kit (CE80545, Chinese Horseshoe Crab Reagent Manufactory Co., Ltd., Xiamen, China). After dilution in sample processing buffer into 1/10 and heated for 10 min at 70°C, plasma LPS content was analyzed following the manufacture's instruction. Plasma insulin and TNF-α concentration were analyzed in 10 μL plasma using ELISA kit (Mercodia, Uppsala, Sweden; DRE30030, China). Plasma levels of lipase, low-density lipoprotein cholesterol (LDL-c), high-density lipoprotein cholesterol (HDL-c), total cholesterol (CHOL), total bile acids, and ALT levels were measured by automation instrument in the Chengdu Public Health Clinical Medical Center.

### Intestinal permeability

The intestinal permeability was assessed through measuring the plasma appearance of 4000-Da fluoresceinisothiocyanate (FITC)-dextran (FD4-1G, Sigma-Aldrich, St. Louis, MO) administered through oral gavage. Briefly, the 6 h fasting mice were administered FITC-dextran (500 μg/kg body wt., 100 μg/ml in PBS) by oral gavage. At 1 h post gavage, 120 μL orbital venous plexus blood was collected and centrifuged at 4°C, 12,000 g, for 8 min. Plasma was diluted with an equal volume of PBS (pH 7.4) and measured the FITC-dextran concentration by a Multiskan Spectrum (Thermo Varioskan Flash) with an excitation wavelength of 485 nm and emission wavelength of 535 nm.

### Histology, immunofluorescence, and immunohistochemistry

Liver sections (5 micron) (fixed in 4% paraformaldehyde and paraffin-embedded) were stained with Hematoxylin/Eosin solution. The intestinal mucus layer was measured with a Periodic Acid Schiff (PAS) red stain kit (ab150680, Abcam), following the manufacture's protocol. For defensins estimation, paraffin-embedded ileum sections (5 micron) were stained with anti-mouse α-defensin1 (provided by Dr. Yoshihiro Eriguchi at University of Southern California). Briefly, deparaffinized sections were rehydrated; the antigen was retrieved with boiled Tris-EDTA buffer for 18 min and blocked with 3% BSA for 1 h at room temperature. Then samples were incubated with antibodies to α-defensin1 overnight at 4°C. After incubation with HRP-conjugated secondary antibodies (ZSGB Bio PV-6000) for 1 h at room temperature, the sections were developed with DAB (ZSGB Bio ZLI-9017), and mounted with Neutral Balsam for analysis under Nikon eclipse Ti-U microscope. Immunofluorescent staining was applied to assess Paneth cells with goat anti-MMP7 (Santa Cruz Biotechnology, Inc., Santa Cruz, sc-8832), followed by secondary fluorescent antibodies (A24431, donkey anti-goat), and DAPI. Slides were mounted with FluoromoutIM Aqueous Mounting Medium (F4680-25 ML, Sigma). Digital imaging fluorescence microscopy of the ileum was performed using a Leica TCS SP5 II system.

### RT-qPCR analysis

Total RNA was isolated using Trizol (Transgen, China) and reverse transcribed into cDNA with PrimeScriptTM RT reagent Kit (TaKaRa, Cat. RR047A). Real-time qPCR contained 2 μl of cDNA, 200 nM primers, and 5 μL of FastStart Essential DNA Green Master (06924204001, Roche) in a final volume of 10 μl; and was performed using Bio-Rad cfx96. Primer sequences are listed in Table 3. Relative mRNA levels were normalized to RPL-19 mRNA expression.

**Table 3:** **Primers used for RT-qPCR analysis**.

**Mouse gene**	**Forward 5′ → 3′**	**Reverse 5′ → 3′**
TNF-α	TGGGACAGTGACCTGGACTGT	TTCGGAAAGCCCATTTGAGT
PAI-1	ACAGCCTTTGTCATCTCAGCC	CCGAACCACAAAGAGAAAGGA
MMP-13	CTTCTGGTCTTCTGGCACACG	GGTAATGGCATCAAGGGATAGGG
RPL-19	GAAGGTCAAAGGGAATGTGTTCA	CCTTGTCTGCCTTCAGCTTGT
PEPCK	ACACACACACATGCTCACAC	ATCACCGCATAGTCTCTGAA
Occluding	ATGTCCGGCCGATGCTCTC	TTTGGCTGCTCTTGGGTCTGTAT
ZO-1	ACCCGAAACTGATGCTGTGGATAG	AAATGGCCGGGCAGAACTTGTGTA
Claudin-2	CCTTCGGGACTTCTACTCGC	TCACACATACCCAGTCAGGC
DEFA5	GCTCCTGCTCAACAATTCTCC	CAGCTGCAGCAGAATACGAA
DEFA2	GGCTCCTGCTCACCAATTCT	GCCTCAGAGCTGATGGTTGT
DEFB1	ACACCCCATCTGCAACCTTA	TGTCCAAGTCCCAACACAGA
CAMP	AGTCTGTGAGGTTCCGAGTGAA	CACCAATCTTCTCCCCACCTTT
MCP1	GCAGTTAACGCCCCACTCA	CCCAGCCTACTCATTGGGATCA
MUC2	GCTCGGAACTCCAGAAAGAAG	GCCAGGGAATCGGTAGACAT
MUC1	CCTTTCTTCCTGCTGCTACTTCT	GGCTGGGTCTGAGTTGCTG
MMP7	GCAGAAGTTCTTTGGCCTGC	TATCCGCAGTCCCCCAACTA

### PCR-based denaturing gradient gel electrophoresis (DGGE) analysis

For PCR-DGGE analysis of the total bacteria, Nested PCR was used to amplify the V2–V3 regions of the 16S rRNA gene. In the first PCR amplification, the universal bacterial 27F and 1492R primers were used to amplify the 16S rRNA gene in a S1000™ thermal cycler (Bio-Rad, USA) using the following program: initial denaturation for 5 min at 94°C, 30 cycles of 94°C for 30 s, 58°C for 45 s, and 72°C for 90 s and final elongation for 7 min at 72°C. The PCR reaction solution (50 μl total) contained 2 μl of DNA template (50 ng/μl), 2 μl of each primer (10 μM), 19 μl of ddH_2_O and 25 μ lof 2 × Taq PCR MasterMix (contained 500 μM dNTP, 0.1U Taq polymerase/μL, 20 mM Tris-HCl, 100 mM KCl and 3 mM MgCl_2_) (TIANGEN, China). Then 16S rDNA was purified using the Gel Extraction Kit (OMEGA, USA) according to the standard protocol. In the second PCR amplification, the universal bacterial HDA1-GC and HDA-2 primers were used to amplify the V2–V3 region of the 16S rRNA gene in a S1000™ thermal cycler (Bio-Rad, USA) using the following program: initial denaturation for 5 min at 94°C, 30 cycles of 94°C for 30 s, 53°C for 30 s, and 68°C for 30 s; final elongation for 7 min at 68°C. The PCR reaction solution (50 μL total) contained 2 μL of DNA template (50 ng/μL), 2 μL of each primer (10 μM), 19 μL of ddH_2_O and 25 μL of 2 × Taq PCR MasterMix (contained 500 μM dNTP, 0.1U Taq polymerase/μL, 20 mM Tris-HCl, 100 mM KCl and 3 mM MgCl_2_) (TIANGEN, China). The sequence of the GC clamp was 5′-CGCCCGGGGCGCGCCCCGGGCGGGGCGGGGGCACGGGGGG-3′.

The PCR products were subjected to DGGE analysis using a 35–65% gradient in 8% acrylamide gel running at 120 V, 60°C for 6 h with the DCodeTM Universal Mutation Detection System (Bio-Rad, USA). After electrophoresis, the gel was stained with SYBR™ Green I (1:10,000 dilution in TAE buffer, Sigma, USA) in dark for 45 min (three times, 15 min each), viewed with a Gel Imaging System (Universal Hood II, Bio-Rad, USA), and photographed. The similarities and differences in the microbial structure were determined by comparing the clusters of the whole DGGE profiles using the Quantity One software package (Version 4.6.9, Bio-Rad, USA). The similarity matrices were produced using the Dice similarity coefficient, which allowed for the construction of dendrograms using the un-weighted pairwise grouping method with mathematical averages (UPGMA) clustering algorithm. PCA was performed (on mean-centered data) to visualize the general structure of species-level composition of gut microbiome using the Canoco for Windows 4.5 Software.

### Gut microbiota analysis by 16S rRNA sequencing

Metagenomic DNA was extracted from frozen ileal lumen contents using a QIAamp-DNA stool mini kit (Qiagen) according to the manufacturer's instructions. The V3–V4 hypervariable regions of the 16S rRNA gene (positions 338 to 806, based on Escherichia coli numbering) were amplified. The forward PCR oligonucleotide contained a 5′ Illumina sequencing adapter, a 10-nt pad sequence, followed by the 338 16S specific linker-primer sequence (5′-ACTCCTACGGGAGGCAGCA-3′). The reverse primer contained the 3′ reverse complement of an Illumina sequencing adapter, the 12-nt Golay barcode, a 10-nt pad sequence followed by the 16S specific 806R reverse linker-primer (5′-CCGGACTACHVGGGTWTCTAAT-3′). PCR reactions were quantified with QuantiFluor™-ST system (Promega Company). Negative controls (no sample added) were included in 16S PCR amplification to test for contaminations. Amplicon pools were sequenced on an Illumina MiSeq sequencer to obtain paired-end 250 bp sequences at the Logic Informatics Company (Shanghai, China). Relative abundance of *Proteobacteria* obtained by analyzing the distribution of OTU sequences (97% level) was expressing as Mean, constructed by Origin 8.5.1 (OriginLab Corporation). Short sequences from 16S rRNA gene were aligned and clustered into 475 OTUs. The resulting OTUs were then matched back to bacteria species. These OTUs were normalized with proportion and squared root arcsine transformation. One-way ANOVA and Student's *t*-test were used to detect differential OTUs in relative abundance under different experimental conditions. Fifty-four OTUs with P values less than 0.05 were selected to construct the heat map (**Figure 5**). Clustergram function in bioinformatics toolbox of MATLAB was used for heatmap creation and bi-clustering. OTUs with similar patterns were clustered together. Different colors on the heatmap indicate OTUS with different relative abundances. As shown on the color bar, where red represents high (normalized) relative abundance, and green shows low relative abundance.

### 16S rDNA PCR analysis

The ileal contents and feces were collected and stored at −80°C before analysis. For microbe analyzing, metagenomic DNA was extracted from frozen ileal content or feces using a QIAamp-DNA stool mini kit (Qiagen) according to the manufacturer's instructions. Q-PCR reactions were carried out with 1 μl of DNA, 200 nM primers, and 5 μl of FastStart Essential DNA Green Master (06924204001, Roche) in a final volume of 10 μl by Bio-Rad cfx96 machine. Relative abundance was normalized to the all bacteria expression. Primer sequences of the microbe are listed in Table 4.

**Table 4:** **Primers of 16S rDNA qPCR analysis**.

	**Forward 5′ → 3′**	**Reverse 5′ → 3′**
All bacteria	ACTCCTACGGGAGGCAGCAG	ATTACCGCGGCTGCTGG
*A. muciniphila*	CAGCACGTGAAGGTGGGGAC	CCTTGCGGTTGGCTTCAGAT
*H. helicobacter*	GCATTTGAAACTGTTACTCTG	CTGTTTTCAAGCTCCCC

### *In vitro* inhibition of *Helicobacter hepaticus* by DEFA5

Original strain of *H. hepaticus* (ATCC 51449) was tested for microbicidal and cytotoxic activities by DEFA5 (Peptides International, INC., USA, PDF-4415) *in vitro*. After activation, the strain was cultured in microaerophilic condition with brain-heart infusion broth at 37°C in an anaerobic jar (Mitsubishi Gas Chemical Company, Inc., Japan) for 5d. Then DEFA5 was added sterilely to the *H. hepaticus* culture at final concentrations of 1, 2, 4, 8, and 16 μmol/L, respectively. After culturing for additional 24 h, the numbers of the bacteria were counted. As the positive control, amoxicillin with clavulanic acid at 2:1 ratio was applied at final concentrations of 10, 20, and 40 μg/mL, respectively. The inhibition ratio of each group was calculated statistical analyzed (*n* = 3), respectively (Table 5).

**Table 5:** **DEFA5 inhibits ***Helicobacter hepaticus in vitro*****.

**DEFA5, mmol/L (mg/ml)**	**X10^7^, CFU before treatment**	**X10^7^, CFU after treatment**	***P*-value**
0	5.92 ± 0.88	5.20 ± 0.63	0.172
1 (0.0053)	4.56 ± 1.28	3.92 ± 0.77	0.367
2 (0.010)	8.08 ± 1.15	7.20 ± 0.63	0.171
4 (0.021)	5.60 ± 0.75	4.16 ± 0.83	0.020
8 (0.042)	6.08 ± 0.59	3.28 ± 0.52	<0.001
16 (0.084)	3.68 ± 0.52	0.96 ± 0.46	<0.001

### Stability of DEFA5 in gastrointestinal conditions

Secreted by Paneth cells in the small intestine as antibacterial peptides in the lumen of intestinal track, alpha-defensins are reported to be persisting throughout the intestinal tract, suggesting that the peptides may mediate enteric innate immunity in the colonic lumen (Darmoul and Ouellette, 1996; Mastroianni and Ouellette, 2009). To calibrate the efficiency of oral gavage of synthetic alpha-defenins 5 we measured its stability through incubation of 1–2 μg peptide with fluid of gastric and small intestine at 37°C for 1 h, followed by Western blot analysis. In such conditions DEFA5 was able to retain over 75% of its intact form.

### Statistical analysis

Microsoft Excel 2003 (Microsoft Corp.) and GraphPad Prism 5.0 (GraphPad Software Inc.) were used for data recording, collection, processing, and calculation. Results were expressed as Mean ± SEM. Differences were assessed using unpaired two-tailed Student's *t*-tests and one-way analysis of variance (ANOVA), followed by Tukey's multiple comparison testing. Statistical significance is displayed as ^*^P < 0.05, or ^**^P < 0.01.

## Results

### Dietary depletion of vitamin D exacerbates the HFD-initiated hepatic steatosis and metabolic disorders

**Figure 1:**
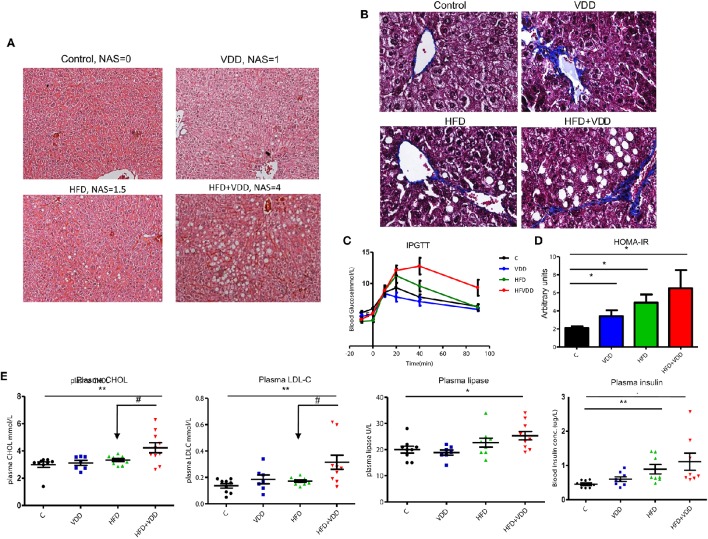
**High fat feeding is not sufficient to induce robust hepatic steatosis and metabolic disorders, but additional vitamin D deficiency is needed**. Balb/C mice were fed for 18 weeks in the following 4 conditions (*n* = 10 per group): (1) control chow, AIN93 with standard VD_3_ supplement at 1000 IU/kg, (2) vitamin D deficient AIN93 chow, VDD, (3) high fat diet (60% calorie from fat) with standard VD_3_ supplement, HFD, (4) vitamin D deficient high fat diet, HFD + VDD. **(A)** Representative images of liver tissues, H&E staining. NAS scores are indicated. **(B)** Representative images of liver tissues, Masson's Trichrome staining. **(C)** Intraperitoneal glucose tolerance test (IPGTT). **(D)** Homeostatic model assessment (HOMA-IR). **(E)** Plasma total cholesterol, low-density lipoprotein (LDL), plasma lipase, and plasma insulin levels in fast. Data represent two independent experiments (*n* = 6–10 mice/group for each measurement). Error bars represent the SEM of samples within a group. ^*^p ≤ 0.05, ^**^p ≤ 0.01 (Student's *t*-test). The arrows indicate the impact of vitamin D supplement to ameliorate the metabolic disorders. ^#^indicates the impact of vitamin D.

**Figure 2:**
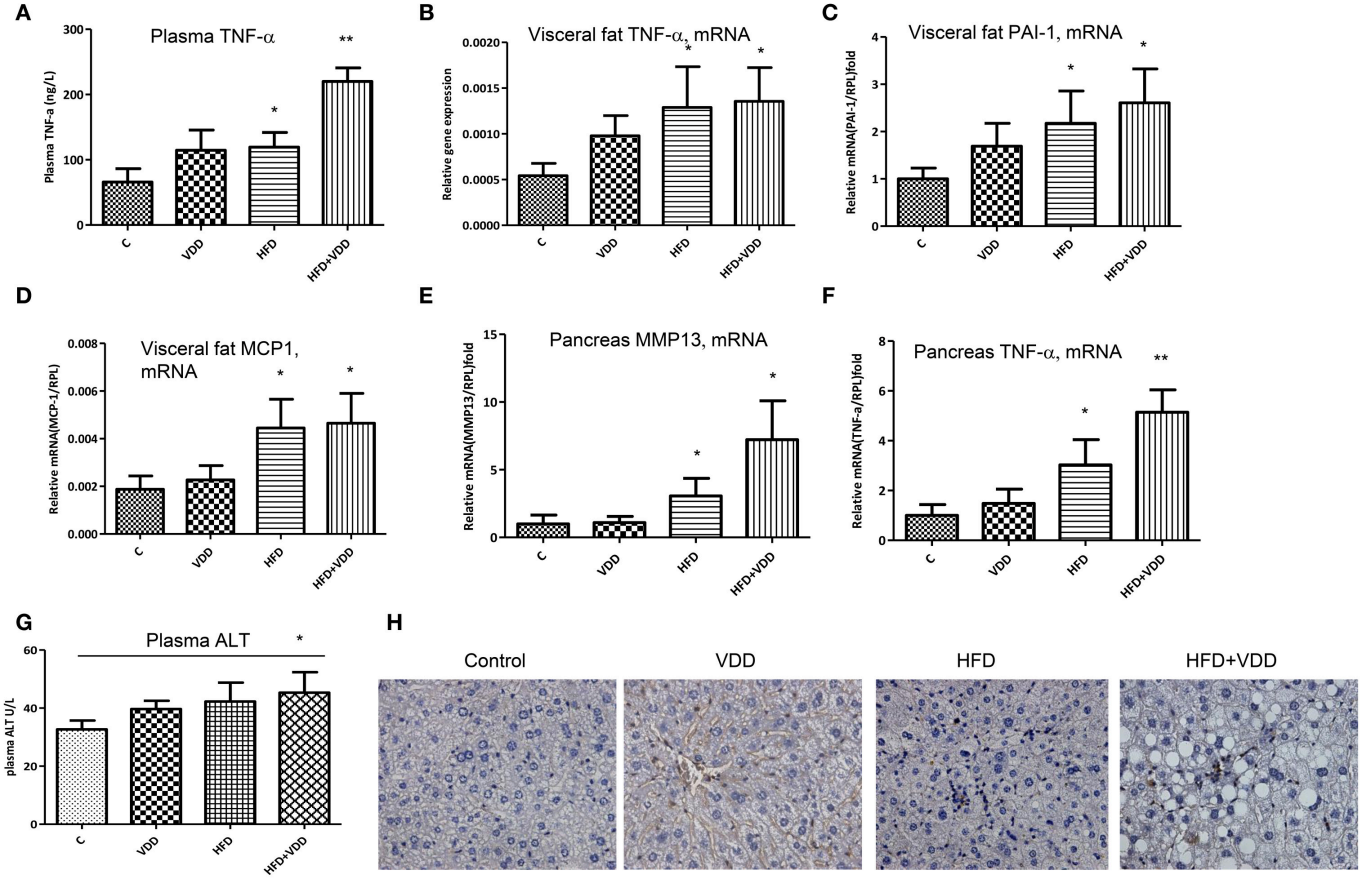
**Systemic and local inflammation is aggravated by dietary vitamin D deficiency**. Balb/C mice were fed with four conditions for 18 weeks as described in Figure 1. **(A)** Plasma TNF-α levels were measured by an ELISA kit. **(B**–**D)** In the visceral fat tissue, the mRNA levels of TNF-α, PAI-1, and MCP-1 were measured by RT-qPCR analysis. **(E**,**F)** In the pancreas, the mRNA levels of MMP13 and TNF-α were determined by RT-qPCR analysis. **(G)** Plasma, alanine aminotransferase (ALT) levels. **(H)** Lymphocyte infiltration in the liver was determined by CD3 staining. Data represent two independent experiments (*n* = 6–10 mice/group for each measurement). Error bars represent the SEM of samples within a group. ^*^p ≤ 0.05, ^**^p ≤ 0.01 in comparison with the control.

BALB/c mice were fed by four types of diet, C for control chow with sufficient VD_3_ supplement (1000 IU/kg), VDD for vitamin D deficient control chow, HFD for high fat diet with VD_3_ supplement, and HFD+VDD for vitamin D deficient high fat diet, (*n* = 10 for each group, and repeating twice), as described in the section of Methods and Materials. After 18 weeks of feeding, the mice fed with HFD or VDD developed moderate hepatic steatosis (Figure 1A, and the NAFLD activity score, NAS). In contrast, the HFD+VDD mice developed severe hepatic steatosis, as indicated by oily ballooned hepatocytes and macrovesicular fat droplets. Pathological significance of NAFLD is its transition from simple steatosis to NASH, featured by inflammation and fibrosis. Liver lesion as indicated by fibrotic septa formation was induced by VDD as we reported recently (Zhu et al., 2015), and here we showed that additional HFD exacerbated the extent (Figure 1B, Masson's Trichrome staining). Likewise, hepatic inflammation was demonstrated by infiltration of CD3^+^ lymphocytes (Figure 2H), and plasma alanine transaminase (ALT) levels (Figure 2G) were evident in the mice subjected to the double hits (HFD+VDD). Importantly, the degree of steatosis was associated with deficiency of plasma 25-OH VD_3_, as described in our previous work (Kong et al., 2014).

Hepatic steatosis more often is a consequence of insulin resistance (IR) and glucose intolerance. In agreement with our expectation, the mice under HFD+VDD feeding generated significant amount of glucose intolerance and insulin resistance, as determined by intraperitoneal glucose tolerance test (IPGTT) (Figure 1C) and intraperitoneal insulin tolerance test (IPITT) (data not shown). VDD in low fat diet did not significantly affect glucose tolerance and insulin sensitivity, but aggravated the HFD-induced glucose intolerance. HOMA-IR analysis showed stepwise escalation of insulin resistance by the mice under VDD, HDF, and HFD+VDD feedings, respectively (Figure 1D). Moreover, plasma lipase and insulin levels were significantly increased on HFD+VDD feeding (Figure 1E), indicating potential pancreatic impairment. Further, HFD+VDD fed mice had elevation of plasma levels of total cholesterol (CHOL) and low-density lipoprotein associated cholesterol (LDL-C), as commonly seen in patients with MetS (Figure 1E). Taken together, our results demonstrate that HFD feeding initiates fatty liver and insulin resistance in mice, but additional VDD worsens the impact and results overt MetS and even NASH, demonstrating the “two hits theory” in a new context.

### Dietary vitamin D deficiency exacerbates the HFD-elicited systemic inflammation, which is critical for insulin resistance and NASH

Systemic and local inflammation, such as that in visceral fat tissue has been causally linked to insulin resistance and MetS (Shoelson et al., 2006; Cefalu, 2009). Indeed, mice fed with HFD+VDD had significantly elevation of plasma TNF-α levels, as compared to the moderate increases in the mice fed with HFD or VDD alone, indicating systemic inflammation by the mice under the double hits (Figure 2A). Expression of inflammatory cytokines (TNF-α, PAI-1 and MCP-1) in the visceral fat tissue was also initiated by HFD feeding (Figures 2B–D). Likewise in the pancreatic tissue, VDD feeding augmented the HFD-initiated up-regulation of TNF-α and MMP13 mRNAs (Figures 2E,F), in agreement with the increased plasma levels of lipase (Figure 1E), suggesting that inflammatory injury might occur in the pancreas by the mice under HFD+VDD feeding. Thus, these findings indicated that lacking dietary vitamin D might exacerbate the HFD-exerted systemic inflammation, which consequently could cause insulin resistance, and NASH formation. Conversely, dietary vitamin D supplement partially but significantly suppressed the efforts of HFD in the induction of metabolic disorders, hepatic steatosis and NASH.

### Vitamin D signaling maintains intestinal integrity

**Figure 3:**
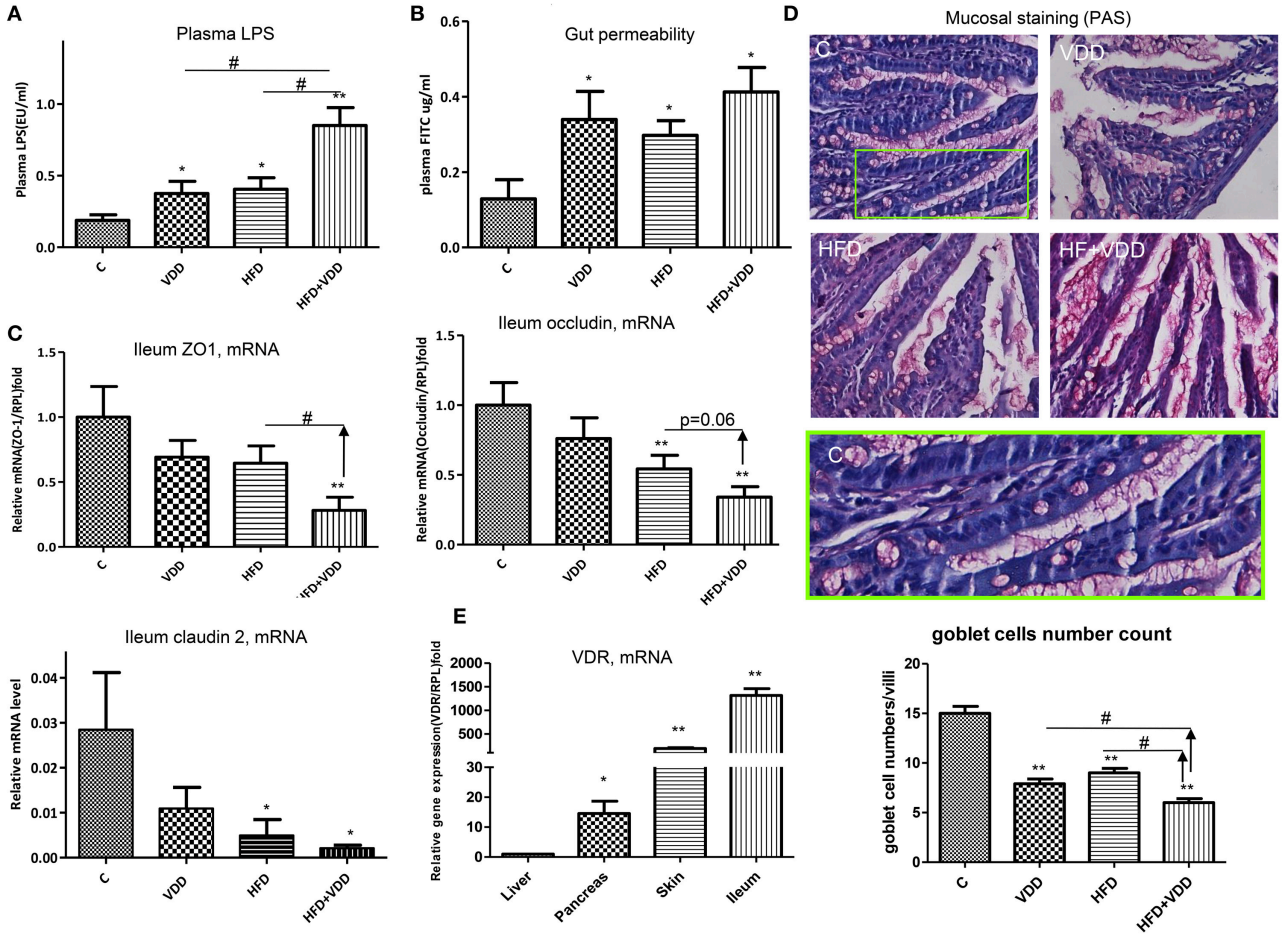
**Vitamin D maintains intestinal homeostasis**. The animal feeding conditions are described as in Figure 1. **(A)** Plasma LPS levels were measured by a Limulus Amebocyte Lysate kit. **(B)** Gut permeability was determined by fluorescein isothiocyanate (FITC)-dextran presented in plasma after oral gavage administration. **(C)** Expression of ZO-1, occluding, and claudin 2 in ileum region was determined by RT-qPCR analysis. **(D)** Periodic Acid/Schiff (PAS) staining. Green box highlights the mucosa and goblet cells in the control. **(E)** Expression of VDR in the ileum, skin, pancreas, and liver by the mice was determined by RT-qPCR analysis. Data represent two independent experiments (*n* = 6–10 mice/group for each measurement). Error bars represent the SEM of samples within a group. ^*^p ≤ 0.05, ^**^p ≤ 0.01 (Student's *t*-test). The arrows indicate the impact of dietary vitamin D on intestinal integrity and plasma endotoxin. ^#^indicates the impact of vitamin D.

Previous work has shown that HFD feeding moderately increased plasma endotoxin and induced insulin resistance (Pendyala et al., 2012), and the latter could be recapitulated by administration of lipopolysaccharide (LPS) to the mice (Cani et al., 2007). Therefore, we asked whether VDD could promote endotoxemia, which consequently exerts systemic inflammation, leading to insulin resistance and hepatic steatosis. In such regard, we found that plasma LPS levels were significantly increased in the mice fed with HFD or VDD, but synergistically elevated in the HFD+VDD mice (Figure 3A). Conversely, dietary VD_3_ supplement significantly attenuated the plasma endotoxin levels, being exerted by HFD feeding. Moreover, the elevation of plasma endotoxin correlated well with the increased gut permeability in these mice (Figure 3B), showing a potential causal relationship. Since tight junctions (TJ) maintain enteral epithelial barrier, and their defects may result in gut impairment, we analyzed the impact of HFD or/and VDD on the key components of the TJ expressed in the ileum. As shown, expression of occludin, ZO-1, and claudin 2 in the ileum regions was moderately suppressed in the mice under VDD or HFD feedings, but synergistically decreased in the mice under VDD+HFD feeding (Figure 3C). Periodic acid/Schiff (PAS) staining of ileum showed that mucosal lattices were apparently detached from the epithelial lining in the mice fed by VDD or HFD, but most markedly in the HFD+VDD mice (Figure 3D). The number of goblet cells indicated by the secretory mucin granules was reduced in the VDD and HFD mice. To determine whether vitamin D signaling plays a role in the maintenance of TJ integrity, we assessed the vitamin D receptor (VDR) expression in the ileum. As shown in Figure 3E, the VDR mRNA is expressed most robustly in the ileum, showing 1300-fold greater than that expressed in the liver, indicating that the ileum, rather than liver, is a major VD targeting tissue in the mice. Likewise, intraperitoneal injection of calcitriol was able to induce 400,000-folds of increase for the ileal *Cyp24A1*, a *bona fide* target downstream of vitamin D-VDR signaling (Supplemental Figure 1). These results suggest a critical role of vitamin D signaling in maintaining the integrity of ileal mucosal lining, in part through maintaining the steady state of the TJ components and mucous proteoglycan (next section).

### Vitamin D deficiency leads to loss of Paneth cell defensins

**Figure 4:**
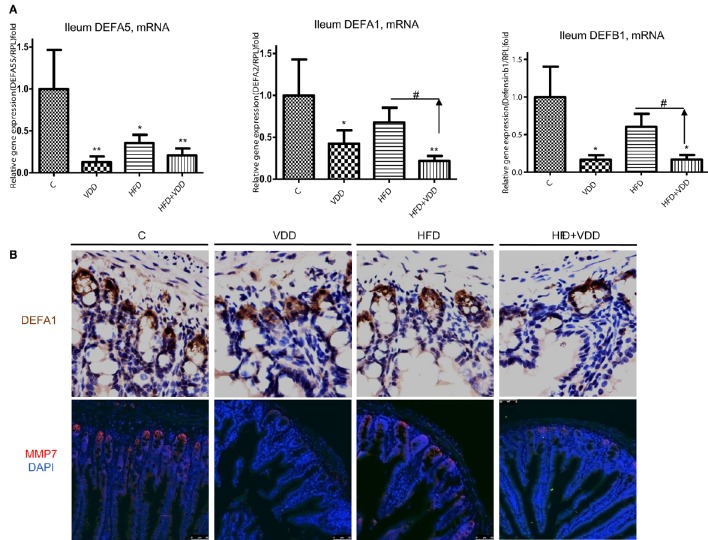
**Vitamin D signaling maintains the steady expression of Paneth cell defensins and their converting enzyme, MMP7**. **(A)** Expression of defensins in the ileum by the mice under the four feeding conditions was determined by RT-qPCR analysis. **(B)** Immunohistochemical staining of the ileal region for DEFA1, and immunofluorescent staining for MMP7 in the crypts of Lieberkühn. Data (*n* = 4–10 mice/group for each measurement) are presented as mean ± standard error. Comparison was conducted by *t*-test between the experimental groups with the control, and ^*^P < 0.05, ^**^P < 0.01. ^#^indicates the impact of vitamin D.

The robust expression of VDR in the ileum and remarkable activation of VD signaling pathway as indicated by Cyp24A1 expression upon calcitriol challenge prompted us to examine the roles of VD signaling in the Paneth cell specific α-defensins, which are secreted into the lumen of gut to balance the gut microbiome population. α-defensin 5 (Defa5) was highly expressed in the ileum of the control mice, as determined by RT-qPCR analysis (Figure 4A). The mRNA levels of the peptide were moderately decreased in the HFD fed mice, but markedly reduced in the VDD and HFD+VDD groups, indicating a critical role of VD signaling in maintaining the expression of the Paneth cell defensins in the ileum. Likewise, the ileal expression of α-defensin 1 (Defa1), as measured by its mRNA and protein in the crypts as well as β-defensin 1 (Defb1) were all decreased in the VDD and HFD+VDD fed mice, again showing vitamin D signaling is critical to regulate the ileal defensins. Furthermore, MMP7, a metalloproteinase that can proteolytically convert pro-α-defensins to their mature and active forms (Wilson et al., 1999) and also serves as a lineage marker for Paneth cells, was fully depleted in the ileal crypts of the VDD and HFD+VDD fed mice as compared to that in the control and HFD groups (Figure 4B). MMP7 protein, as stained in the crypts of Lieberkühn in the control mice, was totally diminished by the VDD and HFD+VDD mice (Figure 4B). These lines of result demonstrated a critical role of vitamin D signaling in maintaining the steady state expression of α-defensins and MMP7 in physiological conditions. In support of this notion, administration of 1,25-dihydroxylvitmain D_3_ to the mice up-regulated defensin mRNA levels in the ileum (Supplemental Figure 1). Lastly, through bioinformatics analysis we recognized putative VDR responsive sites (VDRE, VDR responsive elements) presented within the 5′-promoters of these α-defensin genes (data not shown) suggesting a potential mechanism of direct transcriptional activation in maintaining physiological expression of gut defensins in the condition of sufficient dietary vitamin D supplement. Taken together, these results show the functionality of vitamin D-VDR axis in up regulating and activation/process of ileal α-defensins. Conversely, dietary vitamin D deficiency results in loss of Paneth cell specific α-defensins, which may consequently lead to intestinal dysbiosis and endotoxemia.

### The initial dysbiosis exerted by high fat diet was exacerbated by vitamin D deficiency, showing overgrowth of *Helicobacter hepaticus* and down-regulation of *Akkermansia muciniphila*

**Figure 5:**
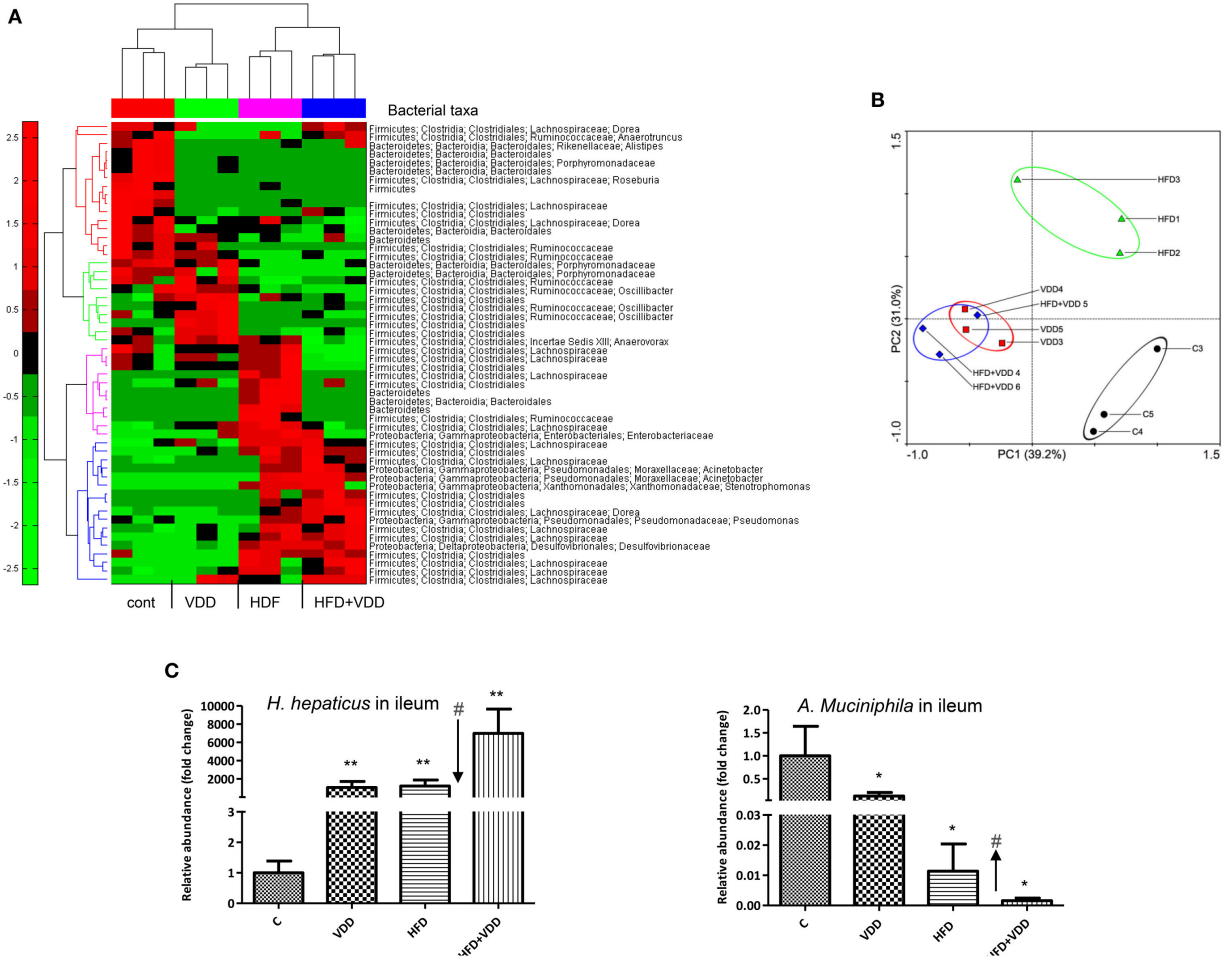
**Initial dysbiosis in the ileum induced by high fat feeding is exacerbated by additional vitamin D deficiency. (A)** The microbiota in the ileal lumen of the mice by the four feeding conditions were determined by 16S ribosomal (rRNA) gene sequencing analysis (*n* = 6), and presented as hierarchical heat-map with bacterial taxa (*n* = 3). **(B)** Principle component analysis (PCA). **(C)** The relative abundances of *Helicobacter hepaticus* and *Akkermansia muciniphila* were determined by qPCR analysis by species-specific primers. Data represent two independent experiments (*n* = 6 for each measurement). Error bars represent the SEM of samples within a group. ^*^p ≤ 0.05, ^**^p ≤ 0.01 (Student's *t*-test). The arrows indicate the impact of vitamin D supplement to ameliorate the dysbiosis. ^#^indicates the impact of vitamin D.

Given the evidence of the ileum as a major VD-VDR targeting tissue and VDR signaling in controlling Paneth cell defensins, we sought to determine the role of vitamin D signaling in maintaining the gut microbiome. We performed 16S rRNA gene high throughput sequencing analysis of the ileal microbiota for the mice under the 4 feeding conditions. More than 90% of the bacteria from ileal contents belong to three phyla, *Firmicutes, Bacteroidetes*, and *Proteobacteria* (Supplemental Figure 2). At phylum levels, the relative abundance of *Firmicutes* was moderately increased, while *Bacteroidetes* was reduced by HFD feeding. Conversely, the phylum of *Proteobacteria* was moderately increased by VDD or HFD feeding. The heat-map with bacterial taxonomy shows the significantly changed ones among the four feeding conditions (Figure 5A). A principal component analysis (PCA) (Figure 5B) shows the enterotypes of ileal bacteria were relatively clustered closely in three distinct patterns: (1) the control, (2) HFD mice, (3) VDD and HFD+VDD mice. Clearly, the phylogenetic clusters of the gut bacteria were progressively changed from the control to HFD, VDD, and HFD+VDD, respectively. We further examined a phylogenetic branch in *Proteobacteria*, showing that the class of epsilon-*Proteobacteria*, the order of *Campylobacterales*, the family of Helicobacteraceae, and the genus of Helicobacter in the taxonomy tree were all increased by VDD or HFD feeding (Supplemental Figures 2B,C, highlighted in red). More importantly, the relative abundance of *H. hepaticus*, which causes hepatitis and liver tumors in certain immune deficient strains of mice (Fox et al., 1994), was found here a big increase in the ileal lumen by the mice under VDD or HFD feeding (Supplemental Figure 2D). On the other hand, the *Akkermansia muciniphila*, a symbiotic in the phylum of Verrucomicrobia was decreased significantly in the ileum of mice fed with VDD or HFD conditions. It was reported that the abundance of *A. muciniphila* is inversely correlated to diabetes and MetS in rodents and humans (Derrien et al., 2004), and probiotic administration of *A. muciniphila* improves MetS in animal models (Everard et al., 2013).

To validate the above findings, we resolved the nested PCR products of the bacterial 16S rRNA genes by denaturing gradient gel electrophoresis (DGGE). More than 40 bands distinctively resolved on DGGE were subjected to sequencing analysis (Supplemental Figure 3). Helicobacter species were identified in multiple hits in the samples of VDD or/and HFD fed mice. We then used qPCR amplification followed by sequencing analysis to confirm the PCR product as *H. hepaticus*, showing a 99% match to the standard ATCC 51449 strain (Supplemental Figure 4). We also quantitated the relative abundances of *H. hepaticus* among the total bacteria presented in the ileal lumen. Results showed that *H. hepaticus* was barely detectable in the ileum of the control mice (5.59 × 10^−5^%), but substantially increased in the VDD, HFD, and HFD+VDD mice, with the relative abundances of 0.081, 0.094, and 0.53%, respectively (Figure 5C). These changes in the abundance of *H. hepaticus* reflected 1000, 1200, and 7000-folds of increases over that in the control mice for the VDD, HFD, and HFD+VDD fed mice, respectively. Conversely, *A. muciniphila* was present in the ileum of control mice at 3.2% relative abundance, but its abundance sharply declined by 8, 82, 626-folds in the VDD, HFD, and HFD+VDD mice, respectively, in comparison with that in the control mice. Importantly, in the ileal lumen of the control mice, the ratio of *A. muciniphila* over *H. hepaticus* was about 100:1, showing predominance of *A. muciniphila* in the control mice. Lacking vitamin D promoted dysbiosis, showing switch the ratio of *H. hepaticus* over the symbiotic *A. muciniphila*. To such regard, we addressed how vitamin D signaling might control the gut eubiosis, and lacking of vitamin D signaling in promoting dysbiosis.

### Oral administration of DEFA5 suppresses gut *H. hepaticus*, restores *A. muciniphila*, and resolves hepatic steatosis

We next investigated whether the overgrowth of *H. hepaticus* was due to suppressed production of ileal α-defensins in the condition of VDD feeding. We first cultured the standard strain of *H. hepaticus* in microaerophilic conditions, followed by incubation with synthetic DEFA5 at different concentrations for 24 h. As shown in Table 5, DEFA5 halted the growth of *H. hepaticus* in a dose-dependent manner, with an MIC of 4 μmol/L (21 μg/ml) and IC_50_ of 8 μmol/L (43 μg/ml), which are within the estimated concentrations of the defensin peptide (HD5) in human intestinal lumen (Ayabe et al., 2000; Wehkamp et al., 2005). Others found MICs for mouse intestinal cryptdins to be ~10 μg/ml for various microbes (Ouellette et al., 1994). Thus, the evidence of direct suppression of the Gram-negative *Proteobacteria H. hepaticus* by DEFA5 supports the concept of a causal link between the impaired expression of DEFA5 and *H. hepaticus* overgrowth (dysbiosis) in the HFD+VDD mice.

**Figure 6:**
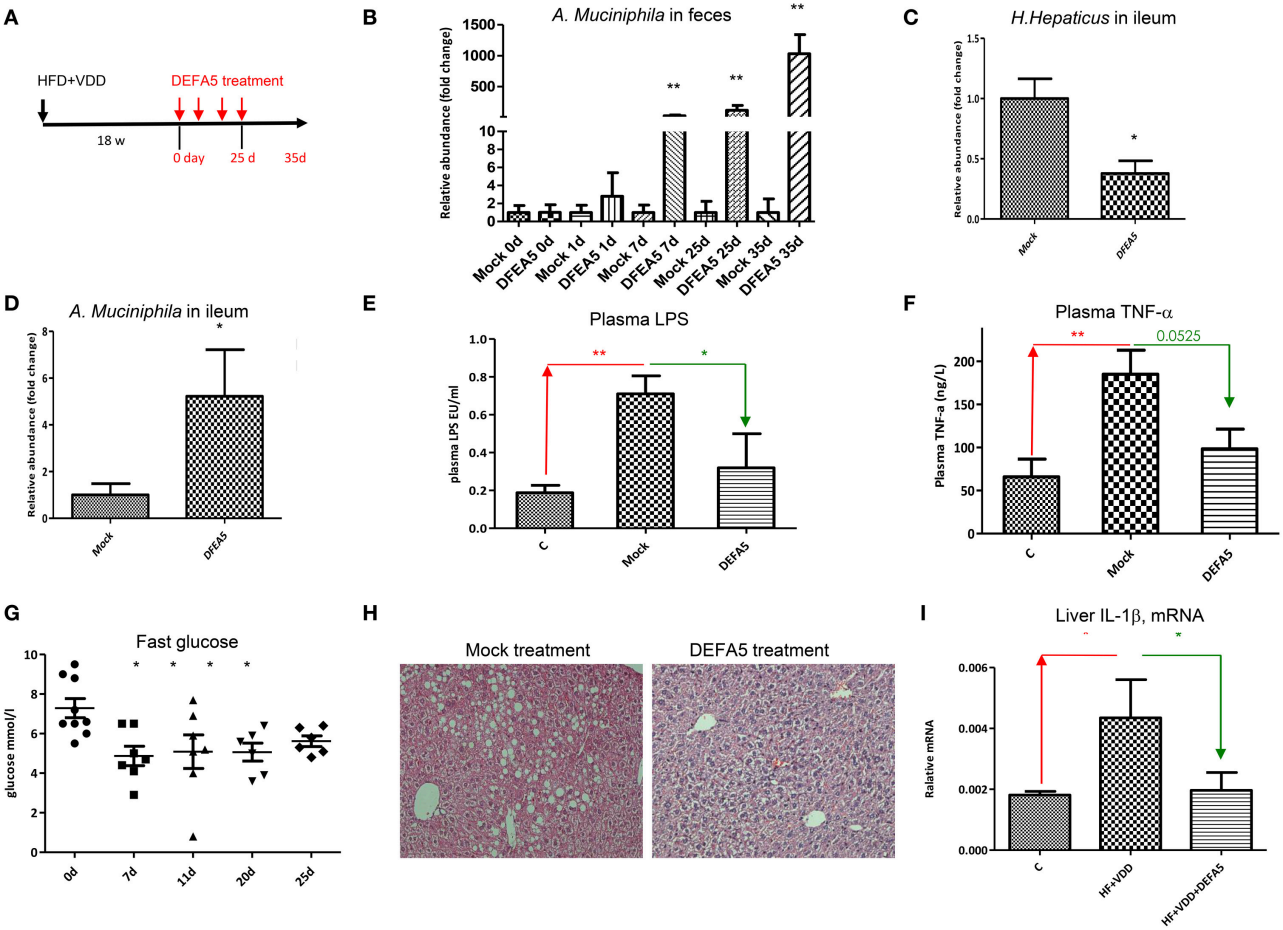
**Oral administration of human alpha-defensin 5 rebalances gut microbiota and resolves hepatic steatosis. (A)** Experimental design. Balb/C mice were fed with HFD+VDD for 18 w, followed by administrated synthetic human DEFA5 (10 μg /dose or mock with saline) by oral gavage for 4 times within 25 d, and the mice were terminated in additional 10 days (*n* = 6 for each group). **(B)** Relative abundance of *A. muciniphila* presented in the feces during the treatments was quantitated by 16S rDNA qPCR analysis. Changing folds over the mock were plotted. **(C)** Relative abundance of *H. hepaticus* in the lumen of ileum after the treatment was quantitated by 16S rDNA qPCR analysis. **(D)** Relative abundance of *A. muciniphila* in the lumen of ileum after the treatment was quantitated by 16S rDNA qPCR analysis. **(E)** The plasma levels of endotoxin after the treatment were determined by Limulus Amebocyte Lysate test. **(F)** The plasma levels of TNF-α was determined by ELISA analysis. **(G)** Fasting plasma glucose during the treatments. **(H)** Representative images of the liver sections, H&E staining. **(I)** The mRNA levels of IL-1β in the liver after the treatment were determined by RT-qPCR analysis. The data (*n* = 6 for each measurement) are presented as mean ± standard error. Comparison was conducted by *t*-test between the experimental groups with the control, and ^*^P < 0.05, ^**^P < 0.01.

We next examined whether oral administration of synthetic DEFA5 would suppress *H. hepaticus* growth *in vivo*. The metabolic disordered mice, generated by HFD+VDD feeding for 18 weeks, were given synthetic human DEFA5 by oral gavage at 10 μg for four times in a period of 25 days (Figure 6A). Two control conditions were applied: the HFD+VDD fed mice received either equal volume of water or the same amount of synthetic mutant DEFD5, by which the conserved Cys and Lys residues were replaced with alanine and serine residues, respectively. During the treatment, the mice were monitored for plasma glucose levels, change of body mass, and fecal microbes. Finally, the mice were euthanized in 10 days after the last gavage treatment. Fecal analysis showed that the relative abundance of *A. muciniphila* was remarkably increased following each of the treatments, reaching 1000-fold greater numbers than that in the control and mock treated conditions (Figure 6B). In the ileum, the abundance of *A. muciniphila* was elevated 5-fold at the end of treatment, while the *H. hepaticus* abundance decreased by 60% (Figures 6C,D).

Remarkably, the suppression of ileal Gram-negative *H. hepaticus* through DEFA5 treatment led to reduction of plasma endotoxin (Figure 6E) and TNF-α levels (Figure 6F), suggesting a linkage. Indeed, insulin resistance was resolved, showing that the elevated fasting plasma glucose levels (Figure 6G) and the body mass (Supplemental Figure 5A) by the HFD+VDD mice were improved through DEFA5 treatment. The hepatic steatosis elevated by the HFD+VDD feeding was also improved by DEFA5 (Figure 6H) along with the reduction of plasma glucose. Hepatic inflammation, determined by the expression of IL-1β mRNA and infiltration of CD3^+^ cells, was attenuated by DEFA5 (Figure 6I and data not shown). Moreover, plasma triglycerides (TG) and LDL-cholesterol levels were also decreased by the DEFA5 treatment, while total cholesterol, HDL-cholesterol, and free fatty acid levels were unchanged (Supplemental Figure 5 and data not shown). The DEFA5 treatment ameliorated steatosis as measured by H&E staining and scoring (Figure 6H). Total plasma bile acid concentration, which is decreased by HFD+VDD feeding and associated with the development of NASH (Kong et al., 2014), was significantly restored by DEFA5 treatment (data not shown) suggesting improved enterohepatic circulation through balancing gut microbiota. Taken together, these results demonstrate that the metabolic disorders induced by VDD+HFD feeding are mediated by the impaired production of intestinal α-defensins, which consequently leads to dysbiosis, endotoxemia, systemic and local inflammation, insulin resistance and hepatic steatosis.

### VDR KO mice exhibit ileal dysbiosis, impairment of mucosa, and hepatic steatosis

**Figure 7:**
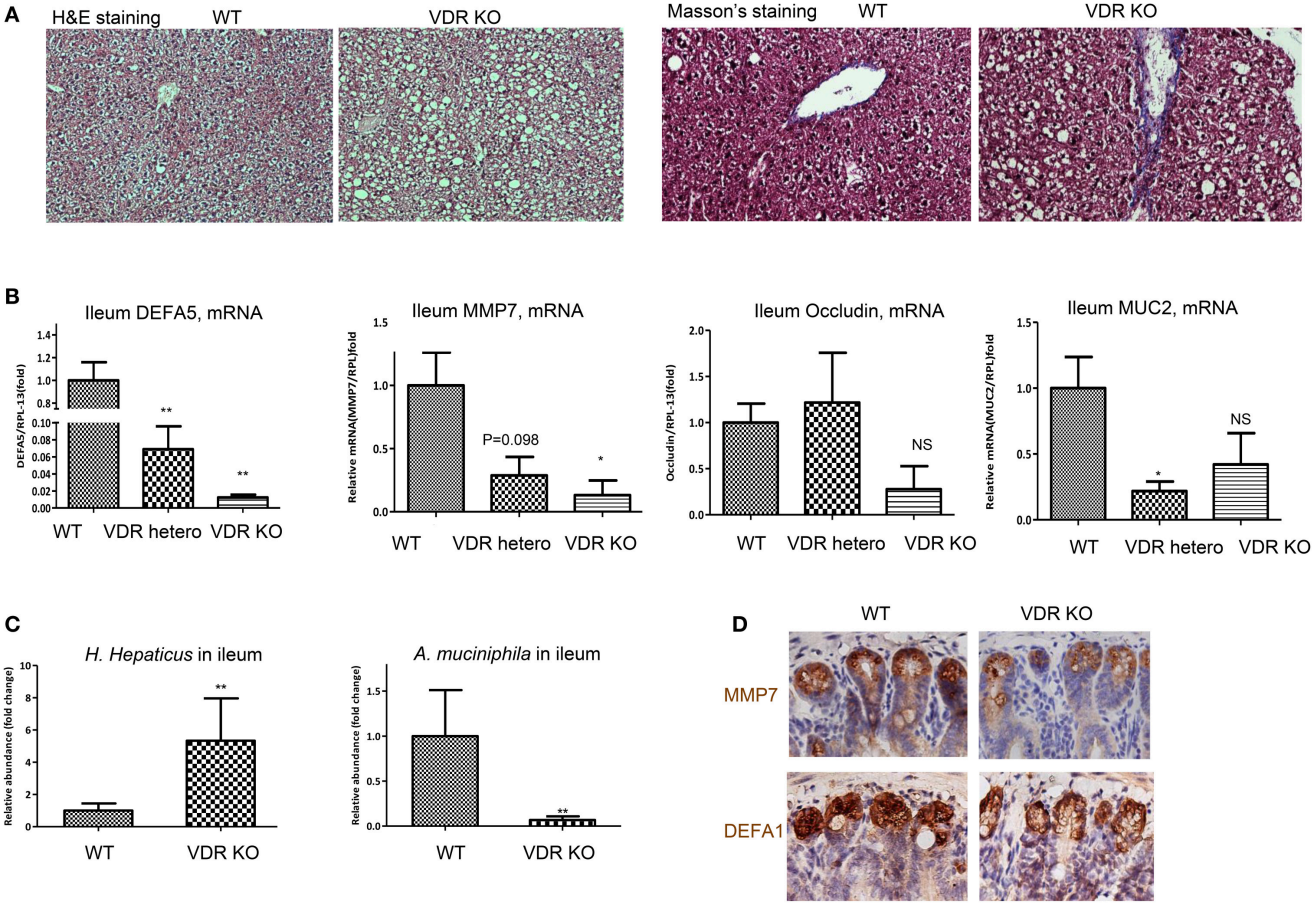
**VDR KO mice exhibit ileal dysbiosis, impaired mucosa, and hepatic steatosis**. VDR KO and WT littermates in the genetic background (B6.129S4) fed with fortified phosphate and calcium chow for 20w were examined. **(A)** Representative images of liver tissues, H&E and Masson's trichrome staining. **(B)** The mRNA levels of DEFA5, MMP7, occludin, and mucin 2 (MUC2) in the ileum of WT, heterozygote, and homozygous VDR KO were determined by RT-qPCR analysis. **(C)** *H. hepaticus* and *A. muciniphila* in the ileum lumen were determined by 16S rDNA qPCR analysis and presented as relative fold of changes. **(D)** Immunohistochemical staining of MMP7 and DEFA1 in the ileum by WT and VDR KO mice. The data (*n* = 4–6 for each measurement) are presented as mean ± standard error. Comparison was conducted by *t*-test between the experimental groups with the control, and ^*^P < 0.05, ^**^P < 0.01.

Finally, using VDR KO mice we measured vitamin D signaling in maintaining homeostasis of intestinal interface and gut microbiota. VDR KO mice are viable, with moderate developmental defects, such as alopecia (Amling et al., 1999) and mild colonic inflammation (Froicu et al., 2006) when maintained on calcium rich diet. As measured by immunohistochemical staining, VDR was highly expressed in the ileum of WT, but diminished in homozygous KO mice (Supplemental Figure 6A). As shown, VDR in the nuclei was richly expressed in the ileal epithelial cells, with the highest levels in the crypts of Lieberkühn of the WT and VDR KO^−/+^ mice. At age of 4–6 months, VDR KO mice developed spontaneous hepatic steatosis (Figure 7A), while body mass was progressively decreased. Liver fibrosis in the VDR KO was previously reported (Ding et al., 2013) and here using Masson's Trichrome staining, we confirmed the issue. Remarkably, expression of DEFA5 and its converting enzyme MMP7, as measured by their mRNA levels in the ileum, were down regulated in the heterozygous and homozygous VDR KO mice with a stepwise manner (Figure 7B), which is in agreement with the results from dietary depletion of vitamin D. Expression of occludin in the ileum was also down regulated in the VDR KO mice. The VDR KO mice had decreased expression of mucin 2 (MUC2), a prominent gut mucosa secreted by epithelial goblet cells. On the other hand, MUC1, which is widely expressed by the apical surface of epithelial cells in lung, stomach, intestines, and the eye, was not significantly altered with VDR KO (data not shown). As expected, the relative abundance of *H. hepaticus* was significantly increased, while the abundance of *A. muciniphila* was drastically decreased in the ileum of VDR KO mice (Figure 7C). PAS staining showed distortion and collapse of mucous membrane in ileum of VDR KO mice (Supplemental Figure 6B), which is related to the aforementioned findings of impaired ileal defensins and dysbiosis of gut microbiota. Finally, using immunohistochemical staining we showed that the expression of α-defensin 1 and MMP7 in the crypts of Paneth cells was all down regulated (Figure 7D).

## Discussion

Epidemiological evidence shows co-prevalence and tight association between vitamin D deficiency/insufficiency and metabolic disorders and NASH (Botella-Carretero et al., 2007; Holick, 2011; Looker et al., 2011; Nelson et al., 2016). Conversely, in animal experiments, artificial sunlight exposure improves NAFLD (Nakano et al., 2011). However, the evidence for the causal role of VDD in the biogenesis of hepatic steatosis and MetS is elusive. In the first part of this work, we show that HFD feeding is necessary, but insufficient to induce robust MetS and hepatic steatosis, and additional VDD as a second hit is needed. Our findings were reproduced in two commonly used mouse strains, BALB/c and C57BL/6 (data not shown). Conversely, dietary vitamin D supplementation in HFD partially but significantly hampered the biogenesis of steatosis and MetS. This finding is consistent with a previous report showing improvement of diabetic outcomes in NOD background mice with a high dose of VD_3_ supplementation (Takiishi et al., 2014).

**Figure 8:**
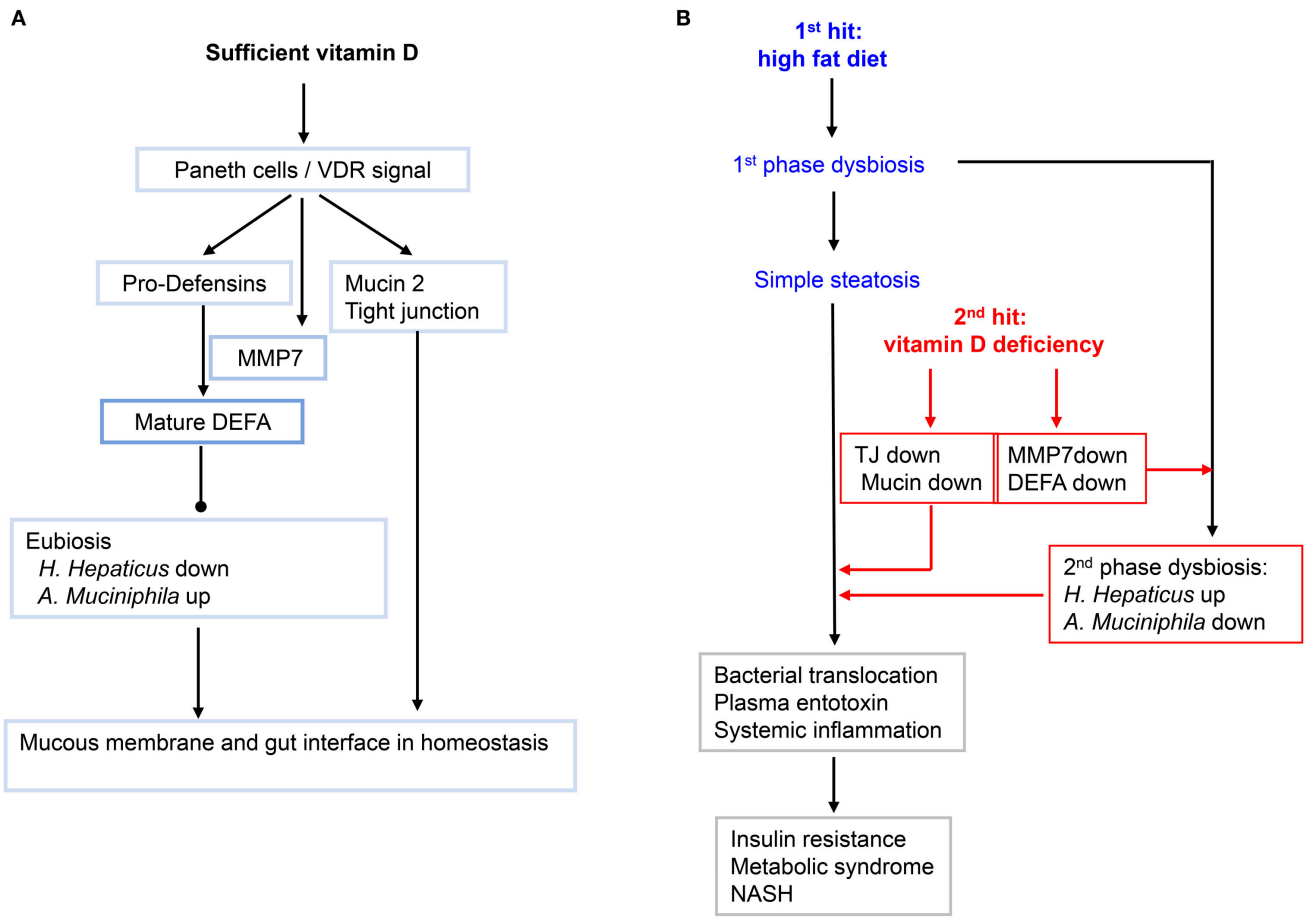
**A working model showing how vitamin D and its signaling in maintain the homeostasis of gut microbiota, and how lacking of vitamin D may exacerbate the high-fat-diet initiated fatty liver and metabolic syndromes. (A)** Sufficient VD and its signaling in maintaining the integrity of small intestine. Gut VDR/Paneth-cells/defensins axis may configure the enterotype of microbiota in eubiosis. Moreover, VD signal up regulates the key components of tight junctions and mucin. **(B)** The primary dysbiosis initiated by HFD is worsened by hypovitaminosis D into second-phase dysbiosis that promotes systemic and hepatic inflammation, leading to insulin resistance, fatty liver and metabolic syndromes, and even transition of simple steatosis and into NASH.

In the second part of this work, we demonstrated a mechanism of vitamin D signaling in controlling the interface of small intestine, defined here as the interplay of the intestinal epithelia and the adjacent gut microbiota. In particular, we showed the key role of vitamin D signaling in maintaining the eubiosis through induction of Paneth cell specific α-defensins (Figure 8A). A previous work showed that vitamin D protects mice from dextran sodium sulfate-induced colitis through regulating the gut microbiome; and conversely dysbiosis as indicated by outgrowth of *Proteobacteria* was evident in the feces of the VDR KO mice under the toxin treatment (Ooi et al., 2013). Using two independent loss-of-function approaches, namely dietary depletion of vitamin D_3_ (VDD) and genetic ablation of vitamin D receptor (VDR-KO), we revealed here a critical role of vitamin D signaling in controlling gut microbiota through optimal expression of Paneth cell specific alpha-defensins and their converting enzyme (MMP7) in the ileum. This notion is further strengthened by the finding that vitamin D signaling in up regulation of tight junction components and MUC2, a prominent mucous proteoglycan made by goblet cells within the ileum. Collectively, these results prompted us to outline a potential pathway for biogenesis of MetS and steatosis under the two hits (Figure 8B). In particular, we showed that high-fat diet with sufficient vitamin D supplement could prevent the biogenesis of MetS and steatosis, in part through maintaining integrity of intestinal interface through up regulation of tight junction components and antimicrobial peptides, which consequently maintain the gut microbiota in a state of eubiosis. Moreover, based on the results we attempted to provide a new working model, in the sense of integrative biology, to explain the biogenesis of avert metabolic disorders and NASH. Importantly, our data showed that typical HFD (with sufficient vitamin D supplement) only generated moderately simple steatosis, in line with mild systemic and local inflammation. And such sense, insulin responsiveness is functional. Also, in such case, the enterotype of gut microbiota showing 1st phase dysbiosis, which is mostly checked through the sufficient levels of alpha-defensins and relatively functional gut tight junction components. Such a notion is demonstrated by the condition of double hits. Under the two hits, the enterotype of gut microbiota is thoroughly altered in a format of deep dysbiosis, called 2nd phase dysbiosis, showing massively increased abundance of *H. hepaticus* in the small intestine and concomitantly decreased population of *A. muciniphila*. In line with a 2nd phase dysbiosis and impaired expression of intestinal components including alpha-defensins as well as increased gut permeability, the plasma endotoxin is significantly elevated to the levels resulting in systemic and local inflammation. The inflammation in the gut, pancreas, and liver is presumably a major driving force for insulin resistance leading to glucose intolerance. As a protective measure, the plasma glucose, which is persistently elevated in the mice under HFD+VDD, is ultimately converted to triglycerides to relieve hyperglycemia. But the persistently elevated hepatic triglycerides in the form of LDL may be further exported to other organs to create metabolic dysorders, on the other hand, the elevated hepatic triglycerides may be stored in the liver as steatosis. It is well known that activation of inflammatory signaling pathways, such as JNK and JAK can block the insulin signaling and GLU4 transport, the basis of insulin resistance in peripheral organs including muscle and adipose tissues (Hirosumi et al., 2002; Nakatani et al., 2004). In the liver, the major function of insulin signaling is to suppress gluconeogenesis and to lower the plasma glucose, while the liver is able to take glucose steadily from the blood supply through steady-state transport by GLU2. Thus, insulin resistance in the liver is featured by loss of insulin-mediated suppression of gluconeogenesis. Whether and how vitamin D signaling engages in gluconeogenesis is largely unknown and is currently under our investigation.

How intestinal dysbiosis is a subject under intensive investigation. Studies showed that while *Bacteroidetes* are decreased in obese individuals as compared to lean ones, *Firmicutes* are increased, and such inverse regulation is served as a key signature for intestinal dysbiosis in MetS patients (Ley et al., 2005). To address the possible mechanism for the intestinal dysbiosis, a previous work demonstrated that transgenic mice expressing human DEFA5 might reduce *Firmicutes* and concomitantly promoted the growth of *Bacteroidetes*, while MMP7 KO mice had opposite consequences, which collectively demonstrated the critical roles of the Paneth cell specific alpha-defensins in maintaining eubiosis (Salzman et al., 2010). Importantly, the impaired expression of the Paneth-cell specific defensins was found in obese human subjects (Hodin et al., 2011). In agreement with these findings, our work demonstrated an axis of vitamin D-dependent expression of DEFA5 and MMP7 in determining gut enterotype of microbiota. This notion is further strengthened by the fact that oral gavage administration of DEFA5 was able to resolve gut dysbiosis. In an *in vitro* analysis, we found that DEFA5 can efficiently suppress the growth of *H. hepaticus*. In line with our work, a recent study showed that θ-defensin RTD-1 improves insulin action and normalizes plasma glucose and FFA levels in diet-induced obese rats (Oh et al., 2015).

Through three complementary measurements, including 16S rDNA deep sequencing, DGGE, and qPCR/sequencing analysis, we found outgrowth of *H. hepaticus* under the conditions of HFD and lacking of vitamin D signaling. Specifically, the outgrowth of *H. hepaticus* was associated with the down-regulation of *defa5* gene, along with impaired expression of other α-defensins. Conversely, oral administration of DEFA5 suppressed ileal *H. hepaticus* and prevented endotoxemia, systemic inflammation and hepatic steatosis in the HFD+VDD mice, revealing a causal role of alpha-defensins in attenuating biogenesis of systemic inflammation, insulin resistance, and hepatic steatosis. Importantly, these results also demonstrated a therapeutic potential of DEFA5 for hepatic steatosis and MetS. Outgrowth of *H. hepaticus* is related to chronic inflammation and neoplasia in susceptible animals (Fox et al., 1994; Avenaud et al., 2003). In murine models, *H. hepaticus* causes chronic hepatitis, IBD, colitis, and colorectal cancer (Ichikawa et al., 2011). *H. hepaticus* was also detected in human bile samples of patients with biliary diseases (Hamada et al., 2009) and is implicated in HCC (Huang et al., 2004; Shimoyama et al., 2010). *H. hepaticus* was initially identified in certain immune deficient mice including IL-10 KO, Scid, and Rag mice, suggesting that impairment of host immune surveillance is a prerequisite for the growth and pathogenesis of *H. hepaticus* (Kullberg et al., 1998). This notion was further supported by findings that *H. hepaticus* infection and induction of colitis relied on host IL-23 and Th17 (Kullberg et al., 2006; Morrison et al., 2013). On the other hand, vitamin D signaling could suppress Th17 cytokine production (Chang et al., 2010), and induce CD4^+^CD25^+^Foxp3^+^ regulatory T cells (Takeda et al., 2010; Hamzaoui et al., 2014). Enterohepatic Helicobacter species including *H. hepaticus* are prevalent in mice from commercial and academic institutions worldwide (Shames et al., 1995; Taylor et al., 2007). Thus, the outgrowth of *H. hepaticus* in the VDD or VDR KO mice is likely related to the impaired immunity, such as lacking regulatory T cells and over activation of Th17 response, which are under our investigation. Whether *H. hepaticus* or other species of Helicobacter directly contributes to NAFLD, T2D, obesity, and MetS in humans should be a subject of investigation.

The symbiotic *A. muciniphila* presents as a highly abundant bacterial species in the feces, inversely correlating with body weight in rodents and humans. Also, oral administration of *A. muciniphila* resolved T2D in a murine model (Shames et al., 1995; Everard et al., 2013). Given our evidence that VDD causes down-regulation of intestinal α-defensins, it is understandable that there would be an outgrowth of opportunistic pathogenic microbes including *H. hepaticus*, but it is unknown as to how VDD causes suppression of the beneficial symbiotic *A. muciniphila*. It is also unknown how DEFA5 administration increases the abundance of *A. muciniphila*. Therefore, there is a possibility that the two species, namely *Akkermansia muciniphila* and *H. hepaticus* may mutually antagonize each other, either directly or indirectly, resulting in two drastically different microbiomes.

## Author contributions

DS, YN, AZ, ZC, PW, LZ, ML, LC, and YL conducted the experiments and acquired data. QS, ZD, SZ, GW, ZX, RH, ZL, and AL analyzed data and critically discussed the manuscript. HT, SP, and YH conceived the work and wrote the manuscript.

### Conflict of interest statement

The authors declare that the research was conducted in the absence of any commercial or financial relationships that could be construed as a potential conflict of interest. The reviewer YK and handling Editor declared their shared affiliation, and the handling Editor states that the process nevertheless met the standards of a fair and objective review.

## Abbreviations

HFD: high fat diet
VDD: vitamin D deficiency or depletion
DEFA: alpha-defensin
DEFA5: alpha-defensin 5
VDR: vitamin D receptor
VDRE: vitamin D receptor response element.
