# Supplementary material for: Vitamin D Signaling through Induction of Paneth Cell Defensins Maintains Gut Microbiota and Improves Metabolic Disorders and Hepatic Steatosis in Animal Models
Source: Front Physiol. 2016 Nov 15;7:498. doi: 10.3389/fphys.2016.00498 (PMC5108805; doi:10.3389/fphys.2016.00498)
Supplement: Supplementary file 1 [file Image1.PDF]

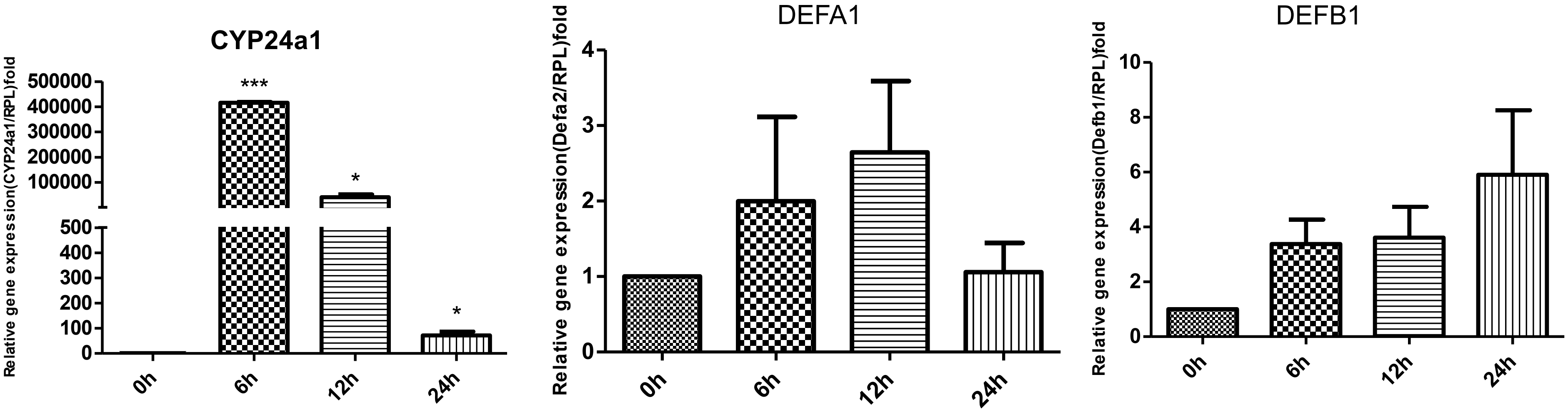

Supplemental figure 1. Ileum possesses a VD/VDR responsive machinery. Balb/C mice under VDD diet for two weeks were given 1,25-dihydroxyl VD_3 for the indicated time periods (n=4-6 for each point). The mRNA levels of Cyp24A1, DEFA1, and DEFAB1 were determined by RT-qPCR analysis. Differences between two groups were assessed using the unpaired one-tailed Student t test. Statistical significance is displayed as * P<0.05, or ** P<0.01.

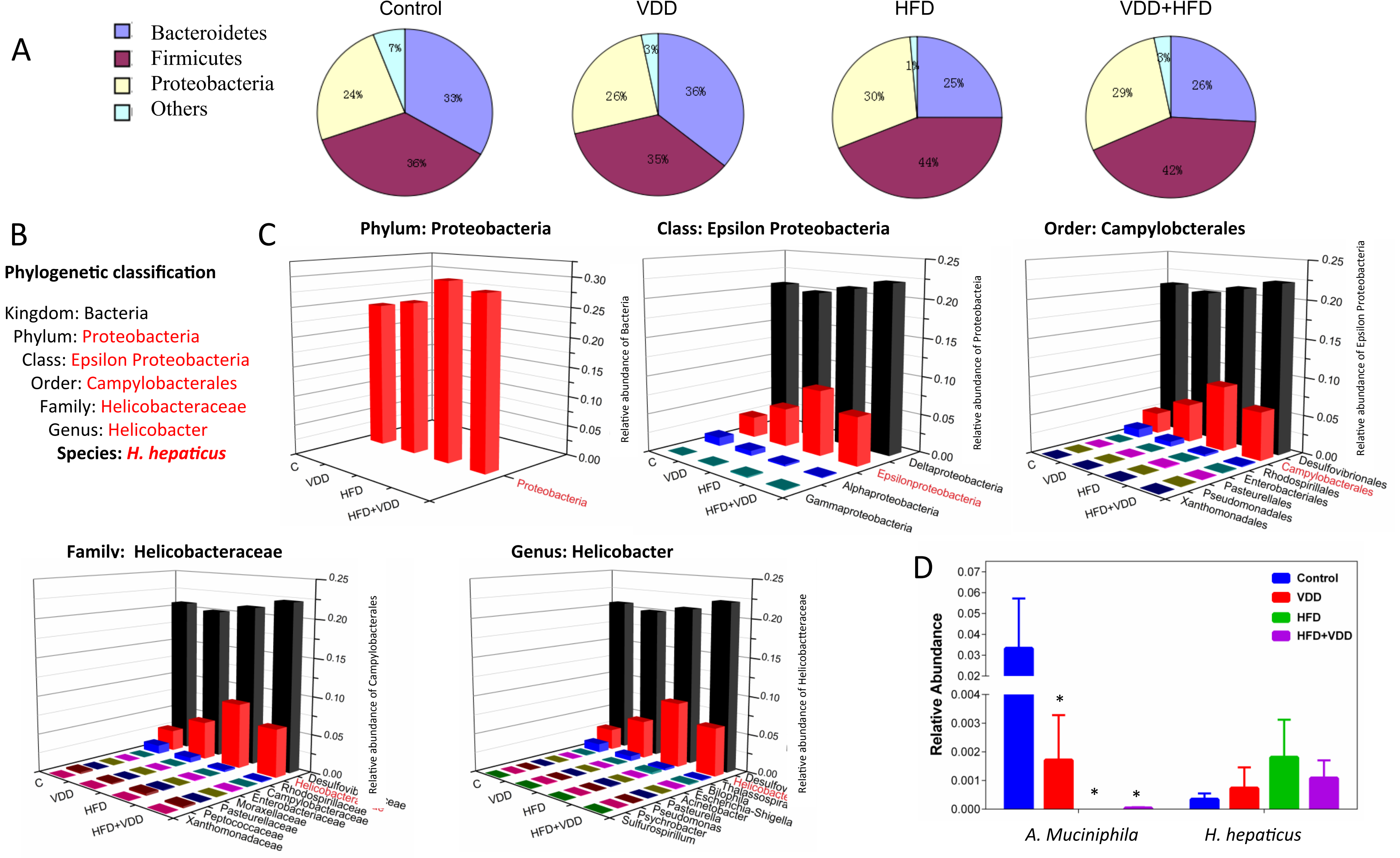

Supplemental figure 2. Changing ileal microbiota under HFD or/and VDD. Balb/C mice were fed with four conditions as described in Fig.1. (**A**) Changing abundances of microbiota at phylum levels in the ileal lumen (n=6). (**B**) Phylogenetic classification of phylum Proteobacteria. (**C**) Abundances of Proteobacteria (phylum), epsilon Proteobacteria (class), Campylobcterales (order), Helicobacteraceae (family), Helicobacter (genus). (**D**)*A. Muciniphila ssp.* of the phylum of Verrucomicrobia and *H. hepaticusssp*.

A

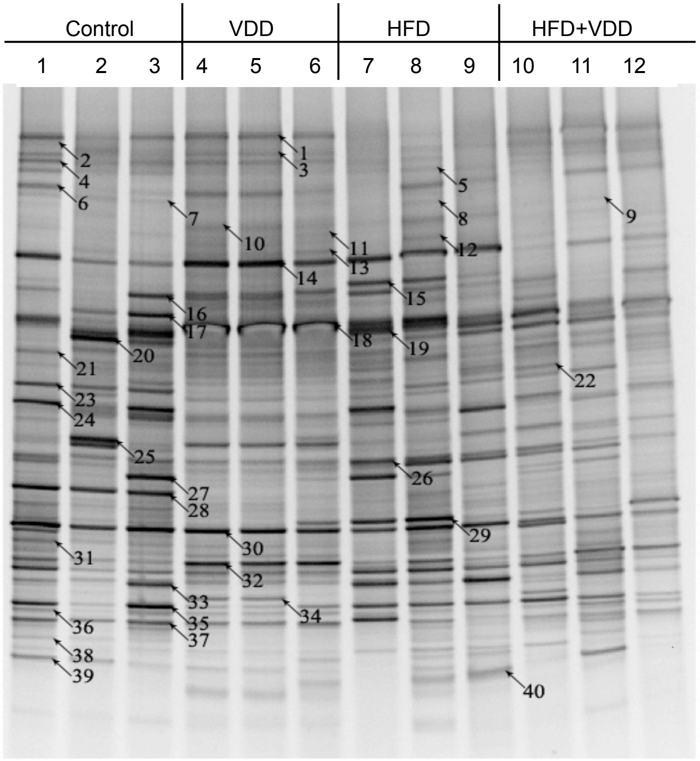

B

Band #; Taxa and similarity

1. Uncultured bacterium clone Pw_B331 (KF842365.1) 94%
2. Uncultured bacterium clone RMAM2207 (HQ321108.1) 83%
3. Bacteroides acidifaciens strain: SLC8-20 (AB599950.1) 99%
4. Uncultured organism clone SBYG_3022 (JN450194.1) 91%
5. Uncultured Cryobacterium sp. clone UCPF29 (KF029471.1) 89%
6. Uncultured Nitrosomonas sp. (HE654678.1) 89%
7. Uncultured **Helicobacter sp**. clone T2118/09_clone4 (HG737518.1) 93%
8. Uncultured bacterium clone ncd171h06c1 (HM261225.1) 87%
9. Odoribacter sp. HGA0030 (JX519710.1) 93%
10. Uncultured bacterium clone 16saw15-01g04.w2k926r (EF603576.1) 95%
11. Uncultured **Helicobacter sp**. clone T2118/09_clone4 (HG737518.1) 93%
12. Uncultured **Helicobacter sp**. clone T2118/09_clone4 (HG737518.1) 96%
13. Uncultured bacterium clone HFDE2698FB01 (JQ893863.1|) 100%
14. Uncultured **Helicobacter sp**. clone T2118/09_clone4 (HG737518.1) 98%
15. **Helicobacter sp.** WBE19 (KF549904.1) 92%
16. Mucispirillum sp. 69 (AB661449.1) 99%
17. Uncultured Deferribacteraceae bacterium (AB700644.1) 99%
18. Eubacterium sp. oral clone JN088 (AY349377.1) 93%
19. Uncultured Deferribacterales bacterium clone: M_Fe_Def01 (AB702920.1) 99%
20. Uncultured rumen bacterium clone: D-C-CA183 (AB616656.1) 99%
21. Uncultured bacterium clone DE06445A08 (JQ892713.1) 97%
22. Uncultured Deferribacterales bacterium clone: M_Fe_Def01 (AB702920.1) 98%
23. Anaerostipes sp. IE4 (AY960568.1) 99%
24. Uncultured Bacteroidales bacterium clone: M_Fe_Bac31 (AB702744.1) 97%
25. Uncultured Bacteroidetes bacterium clone uaP28 (JQ989163.1) 99%
26. Uncultured bacterium clone SWPT20_aaa04a04 (EF097806.1) 99%
27. Uncultured bacterium clone 1.38F (EU655846.1) 93%
28. Uncultured bacterium clone DE06446A02 (JQ892767.1) 98%
29. Uncultured bacterium clone YO00825A07 (KC919852.1) 99%
30. Uncultured Bacteroidales bacterium clone: M_Fe_Bac47 (AB702758.1) 93%
31. Uncultured bacterium clone DE06451F05 (JQ695454.1) 99%
32. Clostridium sp. AL03-15 (FM865974.1) 94%
33. Uncultured Peptostreçptococcus sp. (AM712071.1) 94%
34. Uncultured bacterium clone 16sms289-1a05 (HE607500.1) 99%
35. Uncultured bacterium clone ncd892f05c1 (HM308175.1) 99%
36. Uncultured bacterium clone LUCCWA1007D11 (KC856475.1) 99%
37. Uncultured Clostridiales bacterium clone: M_Fe_Clo008 (AB702849.1) 99%
38. Uncultured proteobacterium clone T5-97 (GU956696.1) 98%
39. Uncultured Lachnospiraceae bacterium clone 4423 (KF505018.1|) 99%
40. Uncultured Bacteroidales bacterium clone: M_Fe_Bac48 (AB702759.1) 98%
41. Uncultured Thiobacter sp. clone CL5.H486 (FM176660.1) 92%

Supplemental figure 3. DGGE analysis of the microbiota in the ileal lumen by mice under HFD or/and VDD feeding. Balb/C mice were fed with four conditions as described in Fig.1. (A) A typical DGGE profile. (B) Taxa identification for the bands labeled in the gel profile. Helicobater sp. were highlighted.

A

Sequence of the qPCR product based on specific primers for *Helicobacter hepacutis*:

GTAGTGGATTCTTGGTGTAGGGGTAAATCCGTAGAGATCAAGAGGAATACTCATTGCGAAGGCGACCTGCTGGAACATTACTGACGCTGATGCACGAAAGCGTGGGGAG
CAAACAGGATTAGATACCCTGGTAGTCCACGCCCTAAACGATGGATGCTAGTTGTTGCCTTGCTTGTCAGGGCAGTAATGCAGCTAACGCATTAAGCATCCCGCCTGGGGA
GTACGGTCGCAAGATTAAAACTCAAAGGAATAGACGGGGACCCGCACAAGCGGTGGAGCATGTGGTTTAATTCGAAGATACGCGAAGAACCTTACCTAGGCTTGACATTG
ATAGAATCTACTAGAGATAGTGGAGTGCCCTTCGGGGAGCTTGAAAACAGA

B

Helicobacter hepaticus ATCC 51449 chromosome, complete genome

Sequence ID: ref|NC_004917.1|**Length:** 1799146**Number of Matches:** 1

Score	Expect	Identities	Gaps	Strand
688 bits (372)	0.0	380/383(99%)	3/383(0%)	Plus/Minus

```
Query  1       GTA-GTGG-ATTCTTGGTGTAGGGGTAAA-TCCGTAGAGATCAAGAGGAATACTCATTGC  57
               ||| |||| |||||||||||||||||||| ||||||||||||||||||||||||||||||
Sbjct  957710  GTAGGTGGAATTCTTGGTGTAGGGGTAAAATCCGTAGAGATCAAGAGGAATACTCATTGC  957651

Query  58      GAAGGCGACCTGCTGGAACATTACTGACGCTGATGCACGAAAGCGTGGGGAGCAAACAGG  117
               ||||||||||||||||||||||||||||||||||||||||||||||||||||||||||||
Sbjct  957650  GAAGGCGACCTGCTGGAACATTACTGACGCTGATGCACGAAAGCGTGGGGAGCAAACAGG  957591

Query  118     ATTAGATACCCTGGTAGTCCACGCCCTAAACGATGGATGCTAGTTGTTGCCTTGCTTGTC  177
               ||||||||||||||||||||||||||||||||||||||||||||||||||||||||||||
Sbjct  957590  ATTAGATACCCTGGTAGTCCACGCCCTAAACGATGGATGCTAGTTGTTGCCTTGCTTGTC  957531

Query  178     AGGGCAGTAATGCAGCTAACGCATTAAGCATCCCGCCTGGGGAGTACGGTCGCAAGATTA  237
               ||||||||||||||||||||||||||||||||||||||||||||||||||||||||||||
Sbjct  957530  AGGGCAGTAATGCAGCTAACGCATTAAGCATCCCGCCTGGGGAGTACGGTCGCAAGATTA  957471

Query  238     AAACTCAAAGGAATAGACGGGGACCCGCACAAGCGGTGGAGCATGTGGTTTAATTCGAAG  297
               ||||||||||||||||||||||||||||||||||||||||||||||||||||||||||||
Sbjct  957470  AAACTCAAAGGAATAGACGGGGACCCGCACAAGCGGTGGAGCATGTGGTTTAATTCGAAG  957411

Query  298     ATACGCGAAGAACCTTACCTAGGCTTGACATTGATAGAATCTACTAGAGATAGTGGAGTG  357
               ||||||||||||||||||||||||||||||||||||||||||||||||||||||||||||
Sbjct  957410  ATACGCGAAGAACCTTACCTAGGCTTGACATTGATAGAATCTACTAGAGATAGTGGAGTG  957351

Query  358     CCCTTCGGGGAGCTTGAAAACAG  380
               |||||||||||||||||||||||
Sbjct  957350  CCCTTCGGGGAGCTTGAAAACAG  957328
```

Supplemental figure 4. Identification *H. hepaticus* ssp. in the ileum of HFD+VDD. (A) sequencing analysis of the PCR products derived from the 16S rDNA of the bacteria in the distal region of small intestine by the mice under HFD+VDD feeding for 18w. (B) Sequence alignment against original strain, ATCC 51449.

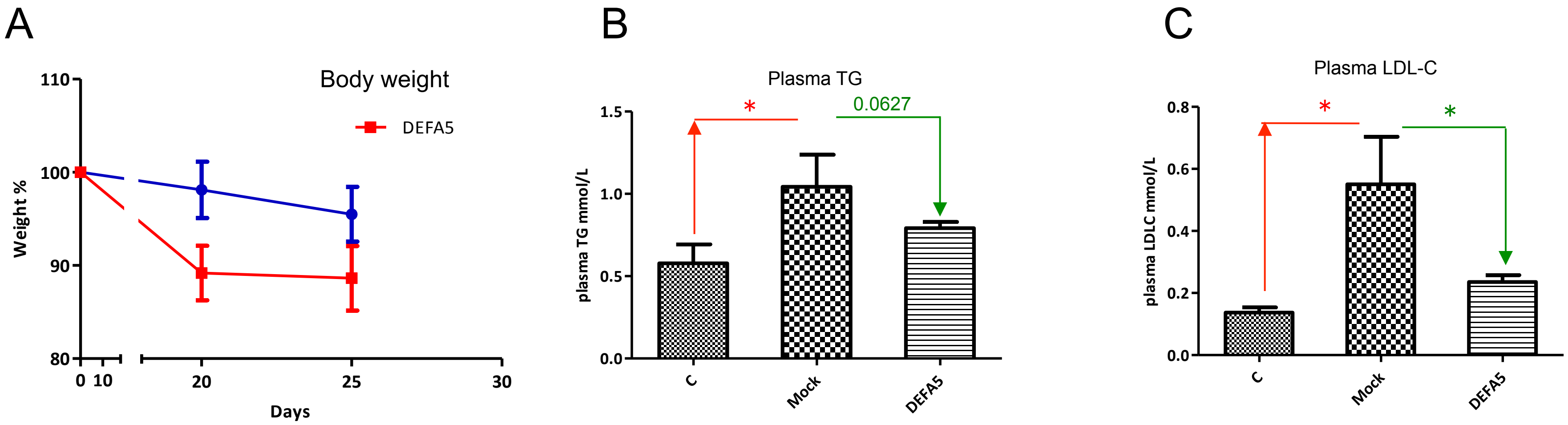

Supplemental figure 5. Oral administration of human DEFA5 improves metabolic disorders in a mouse model. Experimental conditions and treatments were described in Fig.6. (A) Changes of body mass during the treatment. (B) Liver IL-1β mRNA levels after the treatment. (C) Plasma triglycides and LDL-C levels after the treatment.

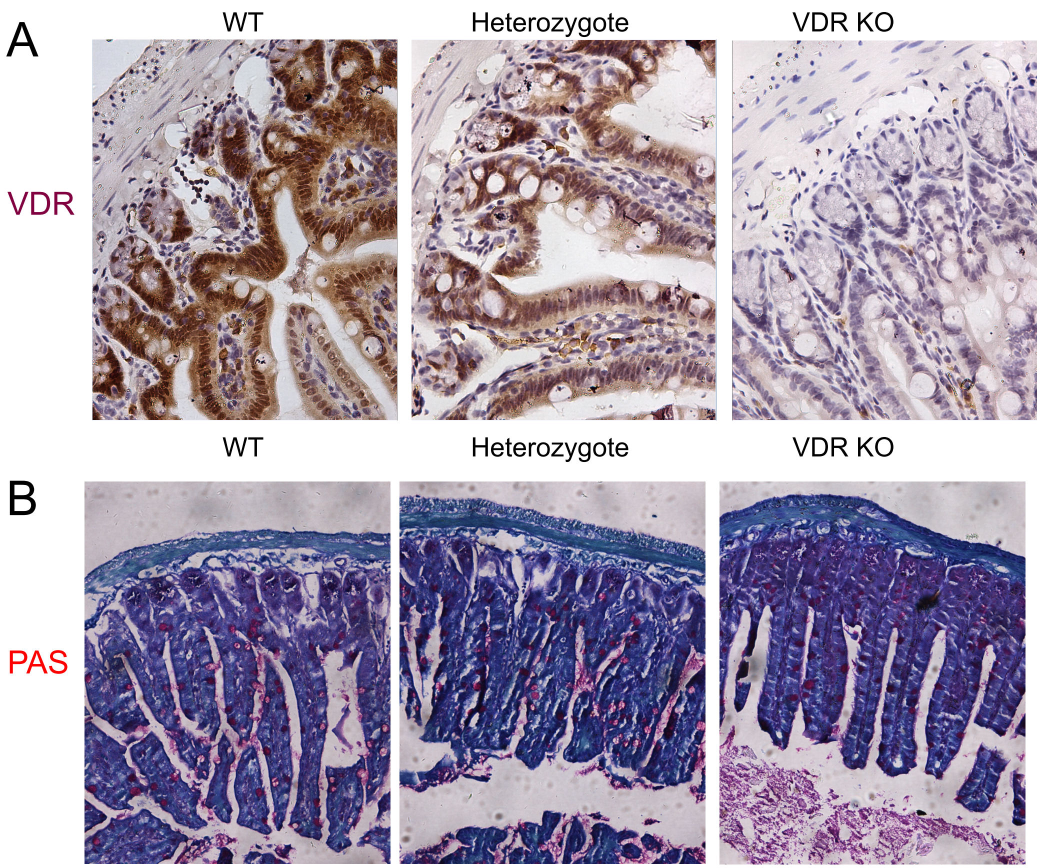

Supplemental figure 6. **VDR KO mice exhibit distorted and collapse of mucous membran**e. (A) Immunohistochemical staining of VDR and (B) PAS staining of the ileum tissues from littermate WT, heterozygote, and homozygote KO.
